# Cross-species functional modules link proteostasis to human normal aging

**DOI:** 10.1371/journal.pcbi.1007162

**Published:** 2019-07-03

**Authors:** Andrea Komljenovic, Hao Li, Vincenzo Sorrentino, Zoltán Kutalik, Johan Auwerx, Marc Robinson-Rechavi

**Affiliations:** 1 Department of Ecology and Evolution, University of Lausanne, Lausanne, Switzerland; 2 Swiss Institute of Bioinformatics, Lausanne, Switzerland; 3 Laboratory of Integrative Systems Physiology, EPFL, Lausanne, Switzerland; 4 University Center for Primary Care and Public Health, University of Lausanne, Lausanne, Switzerland; Center for Genomic Regulation, SPAIN

## Abstract

The evolutionarily conserved nature of the few well-known anti-aging interventions that affect lifespan, such as caloric restriction, suggests that aging-related research in model organisms is directly relevant to human aging. Since human lifespan is a complex trait, a systems-level approach will contribute to a more comprehensive understanding of the underlying aging landscape. Here, we integrate evolutionary and functional information of normal aging across human and model organisms at three levels: gene-level, process-level, and network-level. We identify evolutionarily conserved modules of normal aging across diverse taxa, and notably show proteostasis to be conserved in normal aging. Additionally, we find that mechanisms related to protein quality control network are enriched for genes harboring genetic variants associated with 22 age-related human traits and associated to caloric restriction. These results demonstrate that a systems-level approach, combined with evolutionary conservation, allows the detection of candidate aging genes and pathways relevant to human normal aging.

## Author summary

Aging leads to changes in the activity of genes, but these changes are much more subtle than those between health and disease. This makes finding the genes involved in aging difficult. We have combined two ways to improve information on the involvement of genes in aging. First, important gene activity tends to be conserved in evolution, so we looked for genes whose changes in aging were conserved between distant animal species. Second, genes act in groups, so we looked for changes which affect groups of genes which act together. We were thus able to detect with good confidence subtle changes, which we could confirm with results of experiments modifying diet, and from large genetic scans in humans.

## Introduction

Aging is a process that affects most living organisms and results in a progressive decline in life function and a gain in vulnerability to death [1]. In humans, aging is the main risk factor in a wide spectrum of diseases. The recent increase in human healthspan, also called ‘normal’, ‘disease-free’, or ‘healthy’ aging, is mostly due to improved medical care and sanitation [2,3].

While model organism results are critical to aging research, the identification of evolutionarily conserved molecular mechanisms remains a challenge. On the one hand, broad processes appear conserved, as illustrated by the consistent effect of caloric restriction in extending healthy lifespan in diverse species [4–8]. These common processes have led to the definition of general "hallmarks of aging" [9], such as mitochondrial dysfunction, telomere attrition, or genomic instability. On the other hand, there appears to be very little conservation of orthologous genes in changing expression in aging [10–12]. Thus, although significant efforts have been made to uncover the identity of genes and pathways that affect lifespan, it is unclear to what extent the functional information of aging obtained from model organisms can contribute to understanding human aging. The main model species, fruit fly and nematode worm, are mostly post-mitotic [13–16], whereas most human tissues are proliferative, which affects aging patterns [17,18].

Focusing on the process of aging in healthy individuals should improve the discovery of pathways important in natural aging. Yet this poses an additional challenge to the characterization of molecular mechanisms, as molecular changes influencing healthy aging are much more subtle than those in disease [19]. As gene-level studies are not sufficient to elucidate complex processes such as aging, systems-level analysis of large datasets is an important tool for identifying relevant molecular mechanisms. The integration of various data types contributes to identify pathways and marker genes associated with specific phenotypes [20,21]. Notably, co-expression network analyses can help to elucidate the underlying mechanisms of various complex traits [22,23].

To couple both evolutionary and functional age-related information, we integrated transcriptome profiles of four animal species from young and older adults: *H*. *sapiens*, *M*. *musculus*, *D*. *melanogaster* and *C*. *elegans*. As a source of gene expression, we used human data from the large-scale Genotype-Tissue expression (GTEx) project [24], focusing on two post-mitotic organs, together with aging transcriptomes of model organisms. We identified the functional levels of conserved genetic modifiers important during normal aging, and related them to caloric restriction experiments and enrichments in age-related genome-wide association studies (GWAS). We used gene families as evolutionary information across distant species in a two-step approach to observe age-related conserved mechanisms. Our results show how integrating both network information and evolutionary conservation is informative for a complex phenotype, such as aging. We strengthen the case for a conserved role of proteostasis in normal aging and in the reaction to dietary restriction.

## Results

### Data-driven integrative evolutionary approach to normal aging

**Fig 1:**
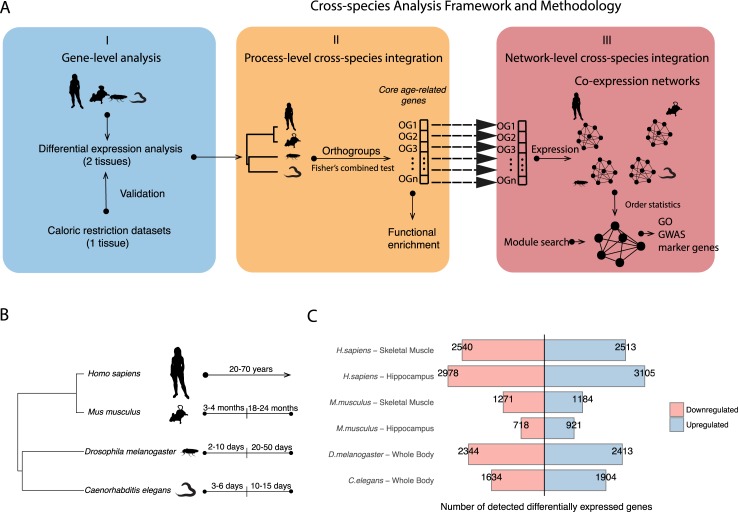
Study design and differential gene expression analysis. (A) An overview of the integration process based on transcriptomes across the species. (I) Analysis starts at the single-gene level by performing differential expression analysis per species between young and old adults (all samples in case of GTEx human data), and determining the orthogroups across species. (II) The orthogroups (OG) are summarized to single genes that represent age-associated conserved genes. (III) The same genes are then used to build the co-expression networks per species and being integrated in the final cross-species network. (See Methods, S1A and S1B Fig) (B) The species used in the study with their phylogenetic relations and the alignment of their ages categories. (C) Barplots representing the numbers of significantly differentially expressed age-related genes (FDR < 0.1) in old individuals in each dataset used. Blue (resp. red) bars represent genes significantly up- (resp. down-) regulated in old adults.

We used an approach in three steps to integrate transcriptomes across distant species (human and model organisms) and to identify evolutionarily conserved mechanisms in normal aging (Fig 1A). In the first step, we performed differential expression analysis between young and old samples in two tissues, skeletal muscle and hippocampus, from humans (*Homo sapiens*) and mice (*Mus musculus*), and in whole body for the fly (*Drosophila melanogaster*) and the worm (*Caenorhabditis elegans*). It was not possible to incorporate sex into the integration of aging effects between species, as data from both sexes were available only in human (S1 Table). We also used transcriptome datasets related to caloric restriction in these species for validation. In the second step, we obtained 3232 orthologous sets of genes, ‘orthogroups’, across those four species (see Methods). Each orthogroup is defined as the set of the orthologous and paralogous genes that descended from a single ancestral gene in the last common ancestor to those four species (*H*. *sapiens*, *M*. *musculus*, *D*. *melanogaster*, *C*. *elegans*) and an outgroup species (*Amphimedon queenslandica*). Each orthogroup can contain a different number of genes, and was treated as a single functional meta-gene common to four species. We corrected for the orthogroup sizes by applying Bonferroni correction on the gene p-values from differential expression analysis within the orthogroup. Then, we selected a representative gene per species within orthogroups. We took the minimum Bonferroni adjusted p-value of a species-specific age-related gene from differential expression analysis. This allowed us to build ‘age-related homologous quadruplets’ (see Details in S1A Fig). The four p-values within each quadruplet were then summarized into a single p-value per quadruplet, by using Fisher’s combined test. We obtained 2511 gene quadruplets in skeletal muscle, 2800 in hippocampus, and 1971 in caloric restriction experiments (S4 Table). We characterized their biological relevance by functional enrichment. In the third and final step, those quadruplets of age-related genes were used to build a co-expression network per species (S1B Fig). These networks were then integrated together using order statistics into one cross-species age-related network. We performed community search algorithm on this network to obtain age-related and evolutionarily conserved modules. The modules were then tested for functional enrichment and for enrichment in GWAS hits.

### Age-related gene expression patterns in four species

To study normal aging, we restricted ourselves to transcriptomic studies with at least one young adult and one old adult time-point, adult being defined as after sexual maturity (Fig 1B). Transcriptomes had to come from control samples (model organism datasets) or from the GTEx dataset (humans), which excludes notably HIV infection, viral hepatitis, and metastatic cancer [25]. While we cannot guarantee that these samples are from healthy individuals, they do represent “normal” aging, as opposed to studies of aging diseases. We defined young and old adults across species as follows: young: 3–4 months for *M*. *musculus*, 2–10 days for *D*. *melanogaster*, 3–6 days for *C*. *elegans*; old: 18–24 months for *M*. *musculus*, 20–50 days for *D*. *melanogaster*, 10–15 days for *C*. *elegans*. For the GTEx data, samples from all adults (20–70 years old) were taken into account in a linear model to detect differentially expressed genes. In human and mouse, we focused on two tissues, skeletal muscle and hippocampus, because they are known to be profoundly affected by aging, and like most fly and nematode tissues are post-mitotic. During aging, skeletal muscle is affected by sarcopenia [26]. Changes in hippocampus function have a significant impact on the memory performances in elderly people [27]. Thus both tissues are susceptible to aging-related diseases. For human, we used transcriptomes of 361 samples from skeletal muscle tissue and 81 samples from hippocampus from GTEx V6p. For the other species we used diverse publicly available transcriptomic datasets (information including sample numbers and data sources in S1 Table). The sample sizes for model organisms were variable, from 3 to 6 replicates per time-point. In order to compare samples between young and old age groups, we fitted linear regression models for each dataset. In addition, in the GTEx dataset we controlled for covariates and hidden confounding factors to identify genes whose expression is correlated or anti-correlated with chronological age, taking into account all samples (see Methods).

We observed uneven distributions of up- and down-regulated genes with aging across different species and datasets (Fig 1C, S2 Table), suggesting variable responses to aging and different power of datasets. The human hippocampus shows substantially more age-related gene expression change than skeletal muscle (6083 *vs*. 5053 differentially expressed genes, FDR < 0.1). However, mouse hippocampus shows less gene expression change than skeletal muscle (1639 *vs*. 2455 differentially expressed genes, FDR < 0.1). These differences are due in part to the smaller sample size of the mouse skeletal muscle study. We limited our analysis to genes that were expressed in at least one age group, leading to detection of 15–40% of genes that exhibits age-related gene expression changes. Of note, these changes are often very small, typically less than 1.05 fold in human and less than 2-fold in animal models.

**Fig 2:**
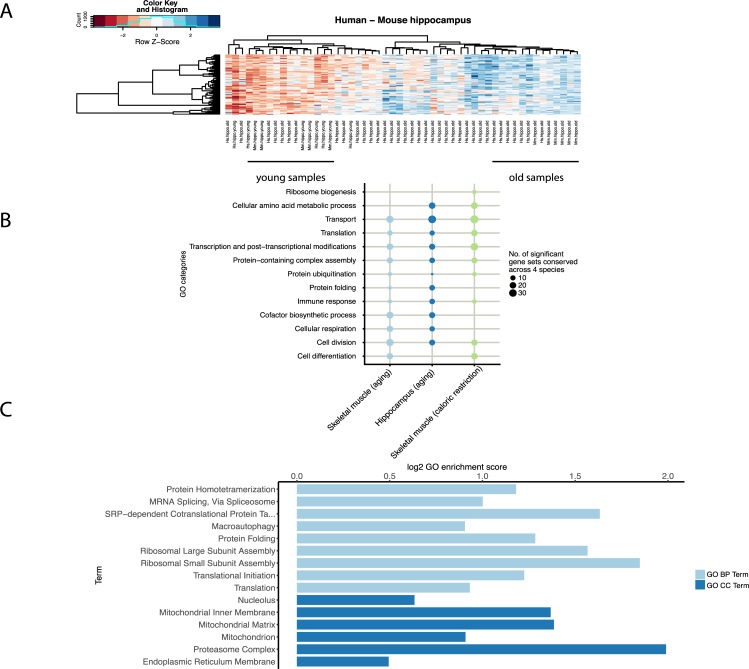
Functional enrichment analysis of integrated age-associated conserved genes. (A) Clustering of the age-related samples between human (20-30y; 61-70y) and mouse. The heatmaps show good concordance between the young and old samples between species based on the 1–1 orthologous genes that are differentially expressed. (B) Bubble plot showing the number of GO categories with conserved change of expression in aging between species. The analysis only includes categorized GO terms that are significant (FDR < 0.05) and unique to the homologous quadruplets enrichment. (C) GO enrichment of genes involved in processes related to proteostasis based on cellular component (CC) and biological process (BP). Lengths of bars represent GO log2-transformed enrichment scores.

It has been previously reported that there is a small overlap of differentially expressed genes among aging studies [28,29]. To make results easily comparable across species, the young and old adults of one species should correspond to young and old adults of another species [30]. Our clustering shows good consistency across age groups of samples between species, based on one-to-one orthologous genes with significant age variation (FDR < 0.05) (Fig 2A, S2 Fig). Yet there is a low overlap of one-to-one orthologous genes with significant expression change in aging (S3 Table). This observation is in line with two studies showing that the overlap between individual genes associated with aging did not reach the level of significance [10,31]. To go beyond this observation, we correlated log-transformed fold change (old/young) between human and model organisms. We observed weak pairwise correlations (S3 Fig) when comparing single genes. This indicates that most transcriptional changes on the gene level are species-specific, and that there is little evolutionary conservation to be found at this level.

### Cross-species integration at the process-level reveals proteostasis-linked age-related mechanisms

To assess the age-related gene expression changes on a functional level per species, we performed gene set enrichment analysis (GSEA) using gene ontology (GO) annotations [32,33]. We then selected GO terms that we grouped into broader categories (S4 Fig). We privileged sensitivity over specificity at this step (FDR < 0.20) to highlight broad trends; thus results concerning individual GO terms should be treated with care. All species showed a general pattern of down-regulation of metabolic processes. The pattern of metabolic down-regulation was stronger in muscle for both human and mouse, while in hippocampus there was down-regulation of nervous system processes. This confirms that there is a tissue-specific signal in normal aging. Due to small samples size of the mouse skeletal muscle dataset, we were able to detect only down-regulated metabolic processes. In addition to metabolism, we observe strong immune systems response to aging in most samples. These results are consistent with known links between metabolism, immunity and aging [34].

We then aggregated processes on the functional level across four species using evolutionary information to observe common age-related mechanisms rather than tissue-specific mechanisms. We integrated differential expression analysis from each species, as described above. We obtained 2010 genes in skeletal muscle / whole body, 2075 genes in hippocampus / whole body, and 1962 genes in caloric restriction experiments (Fisher combined tests, FDR < 0.10). We examined their biological relevance using Gene Ontology enrichment analysis (GEA) based on human annotation. We did not take into account whether the processes that are shared across species are regulated in the same direction, but rather whether they are consistently perturbed during aging.

We obtained 100 significant GO terms (FDR < 0.05) related to biological processes, and aggregated them into broader GO categories. While our species-specific analysis mostly shows tissue-specific pathways, we found that terms with an evolutionarily conserved relation to normal aging are strongly enriched for processes involved in proteostasis, or protein homeostasis. The proteostasis-linked processes showed to be more conserved than expected by chance (S6 Fig). The other conserved processes are related to transport, translation, transcription and post-transcriptional modifications, and protein ubiquitination (Fig 2B, S5 Table). We also confirmed previously known evolutionarily conserved age-related pathways, such as cellular respiration and immune response. Integrating caloric restriction datasets across the four species showed enrichments in similar processes (Fig 2B).

While most of the shared processes have been previously linked to aging, we focused on proteostasis and related processes. To characterize in more detail the specificity of proteostasis-linked processes (Fig 2C), we investigated their enrichment strength in the large human GTEx dataset. Since proteostasis perturbation is detected both through the GO domains of cellular localization and of biological process, we investigate these two domains. We show here the results from the skeletal muscle GTEx dataset, but similar results are observed in the hippocampus GTEx dataset (S6 Fig, S6 Table). The most enriched cellular component terms in skeletal muscle were related to proteasome complex (GO:0000502, enrichment score: 1.99) and to mitochondrial matrix (GO:0005759, enrichment score: 1.38). We also observed strong enrichment of ribosomal large (GO:0000027, enrichment score: 1.57) and small subunit (GO:0000028, enrichment score: 1.84), of protein homotetramerization (GO:0051289, enrichment score: 1.18), and of GO biological processes that are part of the protein quality control network. Overall, the translation and proteasome complexes appear to be the parts of the protein quality control network whose involvement in aging is both evolutionarily conserved across different species, and significant in human normal aging. Interestingly, we also detect mRNA splicing as a part of the conserved processes between species.

**Fig 3:**
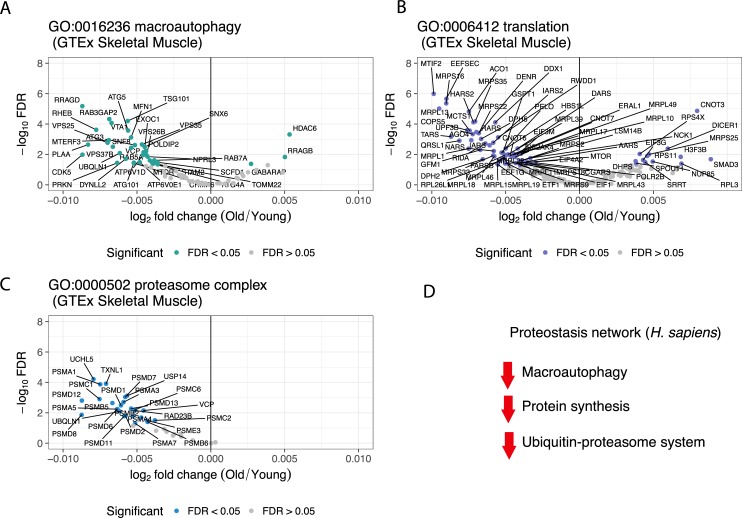
Gene expression changes in the main aspects of the proteostasis network in aging human skeletal muscle. Conserved genes from macroautophagy (A), translation (B) and proteasome complex (C) in GTEx skeletal muscle data. Grey, conserved genes that are not significant (FDR > 0.05) in human GTEx skeletal muscle data. The x-axis of the volcano plots shows the age-regression coefficient across the samples in GTEx data (see Methods; Formula 1), named log2 fold-change. (D) Schematic outline of the gene expression direction of the proteostasis-linked processes in aging human muscle.

The direction of the changes in conserved proteostasis processes in human is consistent with a relation between loss of proteostasis and aging (Fig 3). Although macroautophagy did not show a strong enrichment score in the Fig 2C (GO:0016236, enrichment score: 0.90), there is down-regulation of the conserved genes associated with macroautophagy (Fig 3A), translation (Fig 3B), and the proteasome complex (Fig 3C), which are important in the protein quality network. Similar results are observed in hippocampus, although not with a signal as strong as in skeletal muscle (S7 Fig). The changes during normal aging in both tissues are rather subtle but significant (Fig 3, S7 Table).

### Functional characterization of cross-species age-related network identifies candidate genes related to aging

To characterize age-related processes at a systems-level and to prioritize conserved marker genes associated with normal aging, we constructed probabilistic networks. These were based on prioritization of co-expression links between conserved age-related genes across four species. The conserved age-related genes became nodes in the multi-species network. Thus the connections between those genes will be based on evolutionary conservation, and prioritized according to the their co-expression in each species.

**Fig 4:**
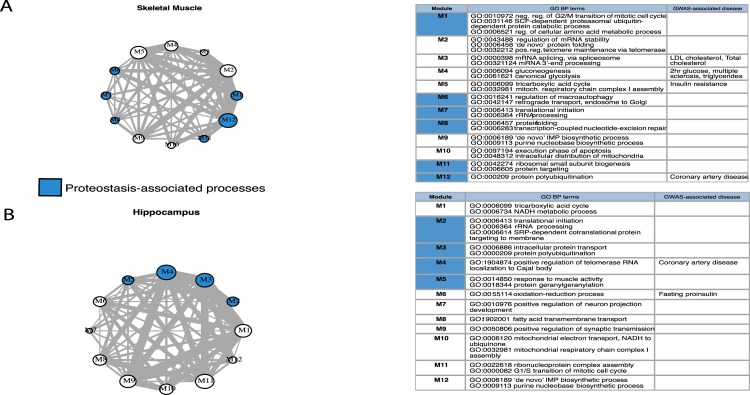
Cross-species aging-associated skeletal muscle and hippocampus functional modules and GO enrichments. Module networks of skeletal muscle (A) and hippocampus (B) with GO and GWAS enrichments for modules of size greater than 10. The top GO term is associated with biological process within the five most significant GO terms (FDR < 0.1). The GWAS-associated disease column contains associations to the module passing a threshold of FDR < 0.2.

Our integrative network analysis initially identified 20 and 14 modules for skeletal muscle and hippocampus, respectively. We randomized our networks 100 times based on the same number of conserved genes per experiment and obtained significantly higher numbers of gene-gene connections than in the original network (permutation test, p = 0.0198) (S9 Fig). Thus aging networks appear to be lowly connected. We focused only on the modules larger than 10 genes; there were 12 such modules per tissue. These modules ranged in size from 16 (M7 hippocampus) to 191 genes (M12 hippocampus) (Fig 4A and 4B, S8 Table). The networks were summarized to module level (module as a node), and we observed strong inter-modular associations. This analysis provided several levels of information. First, it provided a small number of coherent gene modules that represent distinct transcriptional responses to aging, confirming the existence of a conserved modular system. Second, it detected conserved marker genes affected during aging, discussed below.

To determine which of the conserved aging-associated modules are related to the main components of proteostasis network, we carried out functional enrichment analysis on these modules, based on human gene annotations. The enrichments were highly significant for all modules (FDR < 0.01), and confirmed the inter-modular associations (S8 Table). Not all of the modules were related to proteostasis. Interestingly, M1, M10 and M5 in the skeletal muscle network share strong associations with mitochondrion organization and distribution, regulation of cellular amino acid metabolic process and ubiquitin protein catabolic process, while M2 and M3 in hippocampus share associations with different types of protein transport. Other modules (M1, M6, M7, M8, M11, M12 in skeletal muscle; M2, M3, M4, M5, M12 in hippocampus) support the impact of aging on genes related to the proteostasis-linked processes. This included processes related to protein polyubiquitination (GO:0000209), translational initiation (GO:0006413), protein transport (GO:0015031), regulation of macroautophagy (GO:0016241), and proteasome-mediated ubiquitin-dependent protein catabolic process (GO:0043161). In skeletal muscle tissue there were also a strong enrichment in splicing process (M3). Moreover, the connection between M2, M10 and M6 in hippocampus, and between M1, M5 and M12 in skeletal muscle indicates that there is a connection between mitochondrial and proteostasis-related processes. We also performed enrichment analysis based on genes coming from 22 GWAS studies (See Methods, S9 Table). While results should be taken with caution because of the high associated FDR, these modules do appear enriched in metabolic or age-related diseases (Fig 4).

**Fig 5:**
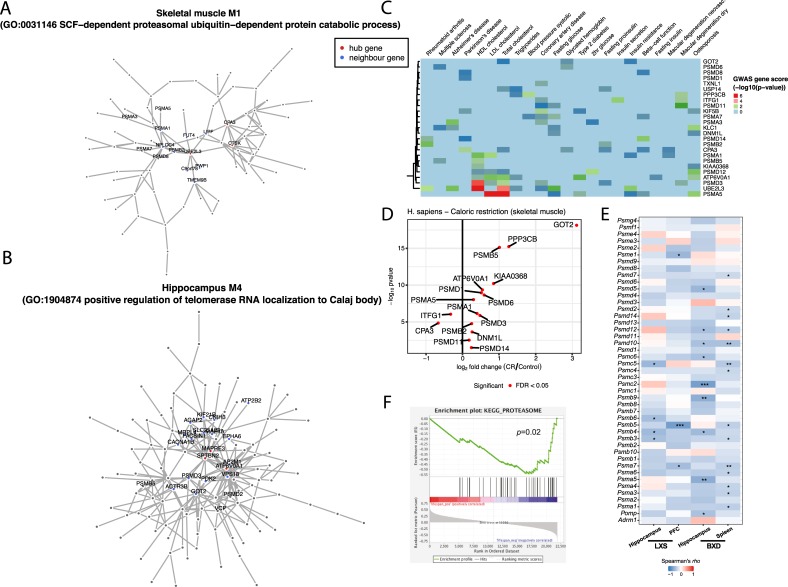
Module architectures and prioritization of candidate genes. (A-B) Architecture of modules related to protein polyubiquitination (M2; A) and positive regulation of telomerase RNA (M4; B) with hub genes (in red) and their neighboring genes (in black) in skeletal muscle. (C) GWAS heatmap of the conserved proteostasis-related genes that were prioritized in modules. The heatmap shows the strength of association of each gene (hubs and neighboring genes from the interested modules) with GWAS. (D) Volcano plot of the prioritized and conserved genes in human dietary restriction dataset. (E) Validation plots for *PSMB5* and other proteasomal genes in the LXS and BXD strains, two independent mouse genetic reference populations. The Spearman correlations between expression values of the genes encoding proteasomal complex and lifespan of the LXS and BXD mouse cohorts are illustrated as heatmap. *, p<0.05; **, p<0.01; ***, p<0.001. (F) GSEA enrichment result between proteasome complex and lifespan.

To further characterize these modules, we studied how conserved modular genes associated with proteostasis and age-related GWAS diseases are changed in expression in humans, as a long-lived species. We looked deeper into the gene composition of two modules, M1 associated with SCF-dependent proteasomal ubiquitin-dependent protein catabolic process (79 genes) and M4 associated with positive regulation of telomerase RNA localization to Calaj body (155 genes) from the skeletal muscle and hippocampus networks, respectively. We defined network hubs, genes that exhibit a significantly high number of connections with other genes in the network, for each of these modules in muscle (Fig 5A and 5B) and hippocampus (S10B Fig). We focused on the hubs with the highest scores in each module and examined their neighborhood. The top ranked genes in M1 of the skeletal muscle were *CTSK*, *UBE2L3 and CPA3* (Fig 5A). They are associated with protein quality network, related to protein degradation. Interestingly, the neighboring genes *PSMB2* and *PSMA1* are associated with the proteasome complex (Fig 5A). The top ranked genes in M4 in skeletal muscle were related to the translational initiation process, with *MAPRE3*, *SPTBN2* and *ATP6V0A1* as hub genes. Their network neighbors were tightly connected to the cytoskeleton and protein transportation (Fig 5B).

Other modules also show links to metabolism and to proteostasis. For example muscle module M12 and hippocampus module M3 are associated with the protein polyubiquitination process (S10 Fig). The top-ranked hub genes in muscle M12 were *DDX3X*, *KIF5B* and *USP7* (S9A Fig). Those genes are related to DNA damage, translation and transport regulations in the cell. In the hippocampus module M3 (S9B Fig), the three hub genes (*PPP3CB*, *DNM1L* and *ITFG1*) are involved in hydrolase activity, apoptosis and programmed necrosis and modulating T-cell function. Although the hub genes with the highest scores were strongly related to metabolism and to tissue-specific functions in each of these two modules, their network neighborhood is associated with the protein quality control network. More specifically, the *PSMB5* and *PSMD3* genes are related to the proteasome complex and are connected to hub genes.

We combined this hub gene analysis with GWAS association gene scores, and observed that *PSMB5*, *UBE2L3*, and *PSMD3* (Fig 5C, S9 Table) are important in many age-related diseases or phenotypes, such as Alzheimer’s disease, HDL cholesterol, LDL cholesterol, triglycerides, and insulin resistance. Other genes related to translation and proteasome complex were also strongly associated to such diseases, such as *PSMB5* with multiple sclerosis (Pascal [35] gene score: *p-value* = 0.0348) and HDL cholesterol (Pascal gene score: *p-value* = 0.0155). Finally, we observed that the prioritized genes associated with age-related diseases from conserved functional modules change in opposite directions with normal aging and with caloric restriction (Fig 5D). This differential expression is consistent with a causal role in these age related diseases, given the attenuating effect of caloric restriction on aging.

### Validation of marker genes using independent mouse studies

We analyzed the association of the expression levels of candidate genes with lifespan in different tissues of mouse recombinant inbred lines used for population genetics analyses, such as the BXD [36,37] and LXS [38] strains. We observed an inverse correlation between lifespan and the transcript levels of not only *PSMB5* but of many genes known to encode proteins of the proteasome complex (Fig 5E) in the hippocampus and spleen of the BXD strains and in the hippocampus and prefrontal cortex of LXS lines. Moreover, the GSEA showed negative correlation between the expression level of genes from the proteasome complex in those tissues and lifespan of the mice (Fig 5F).

## Discussion

The challenge of detecting underlying mechanisms of normal aging that are evolutionarily conserved is thought to be a key impediment for understanding human aging biology [19]. In this work, we integrated evolutionary and functional information of normal (non pathological) aging gene expression to identify conserved age-related systems-level changes. We identified conserved functional modules by integration of co-expression networks, and we prioritized genes highlighted by GWAS of age-related diseases and traits. The observations on several functional levels allowed us to highlight the role of proteostasis, which includes all processes related to protein quality control network, as a strong core process associated with normal aging, at least in post-mitotic tissues.

On the one hand, previous observations restricted to a small number of evolutionarily conserved genes with large effects in aging, or in age-related diseases, provide some evidence that aging mechanisms might be conserved among animals [28]. On the other hand, transcriptome level correlations of expression changes in aging between species are very low in our gene-level results, as in the literature [10–12], which indicates low conservation. Yet the process of aging appears overall conserved, with notably common effects of interventions, such as caloric restriction, showing similar effects across species ranging from nematodes, flies to mammals [8,39]. The solution to this apparent paradox seems to be that pathways are evolutionarily conserved in aging [10], even when single genes are not. Indeed, we have found strong similarities in age-related gene sets between human and other species.

Of note, we restricted ourselves to two post-mitotic organs, and conservation patterns might be different between proliferative human tissues and fly or nematode. The fact that we compared whole body to specific organs remains a limitation of the available data, which we could not correct for. Moreover, we were only able to correct for sex effects on aging in human [40,41]. The mice samples are all male, whereas the fruit fly samples are all female (detailed in S1 Table); nematode worms are hermaphroditic. We might be missing an evolutionarily conserved signal if it is confounded by sex-bias in the available data. Other limitations include the large evolutionary distances and the small number of species sampled, which include only one long-lived species (human). Because of this we might have missed aging signals which are not conserved over bilaterian evolution, or not conserved between short lived and long lived species. A final limitation is that the older model organisms were close to the limit of their life expectancy in captivity, whereas humans of 60–70 years old, while aging, are not at the limit of life expectancy of humans.

Beyond individual pathways, the modular nature of aging has been previously reported at several levels, such as by protein-protein interaction network analysis during human and fruit fly brain aging [23] and blood over human aging [42], human longevity network construction and identifying modules [43], mouse age-related gene co-expression modules identification [44], or aging and age-related diseases cluster detection in human aging [45]. Integrating co-expression networks across species, we identified 10 and 13 evolutionarily conserved functional modules for skeletal muscle and hippocampus, respectively. These conserved modules are not only enriched in processes known to be involved in normal aging, such as immune-related pathways, they significantly overlap with results from age-related GWASs. The latter is all the more interesting that finding causality for aging in GWAS is difficult, given its highly multifactorial nature [46]. Of note, these modules can be tissue-specific, for example related to energy and amino acids in muscle. Thus, aging is an evolutionarily conserved modular process, and this modularity is tissue-specific.

An advantage of our approach is that it allows us to detect with good confidence processes whose changes in aging are quite subtle. This is important because normal aging is not a dramatic process, akin to embryonic development or cancer, but a gradual change in tissues and cell types which keep their defining characteristics. In other words, old muscle and young muscle are very similar at the molecular level, as shown by the log-fold change scale in Fig 3. Yet we are able to detect processes associated to these changes with strong confidence, and these processes are mostly known in to be age-related. The largest changes, thus easiest to detect, include metabolism [47], transcription [48], translation [49], and immune response. Changes in expression for proteostasis related genes are weaker, yet integrating at a systems-level between species provided us with a strong signal.

More broadly, our results strengthen the case for further investigation into the molecular program that links proteostasis to normal aging. Indeed, one of the major hallmarks of aging is the loss of proteostasis. Loss of proteostasis is related to major human pathologies, such as Alzheimer’s and Parkinson’s disease, offering an opportunity to detect conserved candidate genes important in those age-related diseases [50]. The proteasome complex, one of the three major mechanisms of proteostasis [51], is maintained throughout the life of the long-lived naked mole rat [52]. Perturbation of components of proteostasis has been shown to extend the lifespan of mice [53]. These observations on short and long lived rodents are consistent with a role of proteostasis in lifespan of these species [54]. Finally, caloric restriction, defined as a reduction of regular caloric intake by 20–40%, extends lifespan and delays the onset of age-related diseases in many species [4,6–8,55,56], in part through effects on proteostasis networks. Moreover, “loss of proteostasis” is one of the nine proposed hallmarks of aging [9,57]. It is possible that this marker is stronger because of our focus on aging in post-mitotic organs. Aging involves a deregulation of the protein quality control network, and this is conserved between distant species. Changes in protein synthesis and protein degradation processes of proteostasis system may be fundamental to the response to normal aging because the accumulation of somatic and germline mutations can alter fine modulation of the protein homeostasis network and produce pathological alterations. Thus proteostasis provides a link between somatic genome-level changes and the phenotypic impact of aging. During normal aging, the alterations in proteostasis networks are rather subtle and discrete, by contrast to the strong down-regulation of metabolic processes. This suggests that perhaps there is a cascade of triggered pathways as aging proceeds. Moreover, we detect evolutionarily conserved links inside modules between mitochondrial deregulation (hub genes) and protein homeostasis (neighboring genes) in normal aging, consistent with a recent report [58].

The main evolutionarily conserved gene candidates from proteostasis, *PSMB5* and *PSMD3*, are related to the proteasome. These two genes were tightly connected to metabolic hub genes in skeletal muscle and to filament organization genes in the hippocampus. The proteasome complex is down-regulated during aging in our results, and in a transgenic mouse mutant proteasome dysfunction led to shorter lifespan [59]. Moreover, both genes showed significant association in GWAS studies with metabolic and disease traits. Although only the *PSMB5* gene was an experimentally validated candidate gene in mice, the *PSMD3* gene was related with coronary artery disease, HDL cholesterol and fasting proinsulin, and would also be worthwhile to explore further.

The association with caloric restriction studies strengthens the relevance of the processes we report. We observed that gene-set signal was both evolutionarily conserved in caloric restriction, and shared between normal aging and caloric restriction experiments. Genes related to proteostasis change expression in opposite directions between human aging and caloric restriction. This indicates that these functions are maintained during caloric restriction in humans, and strengthens the case for a causal link between proteostasis and normal aging. Our observations are consistent with previous research in *C*. *elegans*, reporting improvement of proteostasis during caloric restriction treatments and extension of the lifespan [60,61]. Notably, *PSMB5* and *PSMD3* follow this trend in caloric restriction relative to aging, further suggesting that they are prime candidates to study transcriptional regulators underlying functional modules in normal aging.

Integrating biological processes based on evolutionary conservation allows distinguishing relevant signals from noise, despite the weak patterns in aging transcriptomes. Moreover, the fact that a same process is involved in aging in very different species strengthens the case for causality. This provides a promising foundation to search for relevant biomarkers of healthy aging of specific tissues.

In summary, the large-scale, comprehensive gene expression characterization in our study provided insights in underlying evolutionarily conserved mechanisms in normal aging. While metabolic and certain tissue-specific pathways play a crucial role in aging, processes affecting the protein quality control network show weaker but very consistent signal. Using both evolutionary and functional information, we detected evolutionarily conserved functional modules allowed us to identify core proteostasis-related genes. These genes were implicated as important hits in age-related GWAS. Together, the integrative systems-level approach facilitated the identification of conserved modularity of aging, and of candidate genes for future normal aging biomarkers.

## Materials and methods

### Data selection

To obtain a representative set of aging gene expression experiments, a set of raw RNA-seq and microarray datasets of four species (*H*. *sapiens*, *M*. *musculus*, *D*. *melanogaster*, *C*. *elegans*) were downloaded from the GEO database [62] and SRA database [63] (S1 Table). For observing aging gene expression signatures in human and mouse, we selected hippocampus and skeletal muscle tissues. The aging gene expression experiments for fly and worm were available as whole-body experiments. All the normal or control samples came from two extreme age groups (young and old adults) that are counted from sexual maturity. This corresponds to 20–30 years old humans, 3–4 months old mice, 4–5 days old flies and 3–6 days old worms (see Fig 1B) in young adults. In old adult age group, this corresponds to 60–70 years old humans, 20–24 months old mice, 40–50 days old flies and 12–14 days old worms. The sample size per age group was 3–6 replicates. The GTEx V6p read counts were used as *H*. *sapiens* aging experiment (V6p dbGaP accession phs000424.v6.p1, release date: October, 2016). The information about the sample ages was obtained through dbGAP annotation files of the GTEx project (restricted access). Two RNA-seq datasets were matched for *M*. *musculus* and *C*. *elegans*; and the microarray platforms included were from Affymetrix: Mouse 430 A/2.0, GeneChip Drosophila Genome array and *C*. *elegans* Genome array.

### GTEx v6p analysis

From the downloaded GTEx V6p data, we extracted the gene read counts values for protein-coding genes by using Ensembl (release 91). For each tissue, the lowly expressed genes were excluded from data analysis according to the GTEx pipeline [24]. Prior to the age-related differential expression analysis, we used the PEER algorithm [64] in a two-step approach to account for known covariates as well as for hidden factors present in GTEx V6p data per tissue. From covariate files (Brain_Hippocampus_Analysis.covariates.txt and Muscle_Skeletal_Analysis.covariates.txt), we used information about the three genotype principal components. From phenotype file (phs000424.v6.pht002742.v6.p1.c1.GTEx_Subject_Phenotypes.GRU.txt), we used information about age, gender, ischemic time and BMI information. From attribute file (phs000424.v6.pht002743.v6.p1.c1.GTEx_Sample_Attributes.GRU.txt), we extracted information about the sample associations with interested tissues, hippocampus and skeletal muscle. In the first step, the PEER algorithm discovers patterns of common variation; it created 15 and 35 assumed global hidden factors for hippocampus and skeletal muscle, respectively. In addition to global hidden factors, we provided age, BMI, sex and ischemic time as known covariates in PEER model. In the second step those hidden factors (gene expression principal components) that showed significant Pearson’s correlation coefficient with age (p-value < 0.05) were excluded. The number of hidden factors that did not significantly correlate in hippocampus was 7/15 and in skeletal muscle were 22/35 that were selected for further linear model analysis. The sum of remaining hidden factors and known covariates were included in a linear regression model to obtain the genes differentially expressed during age in GTEx V6p data for each tissue (Formula 1).

$$Y_{ji}=\mu_{0}+\alpha_{j}{Age}_{i}+\gamma_{j}{Sex}_{i}+\beta_{j}{BMI}_{i}+\theta_{j}{Ischemic\;time}_{i}+\sum_{k=1}^{n}\delta_{j}{PC}_{ki}+\epsilon_{i}$$ [1]

where, Y_ji_ is the expression of a gene *j* in a sample *i*, where *Age*, *Sex*, *BMI*, *Ischemic time* of sample *i*, with their regression coefficients *α*, *γ*, *β*, *θ*. *PC*_*ki*_ (1 < k < *n*) is the value of the *k*-hidden factors for the *i*-th sample with regression coefficient δ; *n* is a total number of factors that was not correlated with age, *ε*_*i*_ is the error term, and *μ*_*0*_ is the regression intercept. If *α* > 0, gene *j* was treated as up-regulated, otherwise gene *j* was treated as down-regulated. The linear model (Formula 1) was performed in *limma voom*, and the p-values were corrected for multiple testing by performing false discovery rate (FDR) correction using Benjamini-Hochberg method.

### Statistical thresholds

Across this study, we have not fixed one threshold of p-value or of FDR (false discovery rate). One can select entities, such as genes, based on any arbitrary FDR threshold depending on what is done with the selected set. When treated as a group one can make statements about properties of the selected entities, for example enrichment analysis (e.g. [65]) or use them for prediction (e.g. Sup. Fig 10 of [66]). When the selected set of entities is not the end-point of the analysis, but is statistically tested for a property, any threshold can be applied and the enrichment test has to be controlled for type I error (or FDR). An advantage of the FDR is that its interpretation relative to a list of results is intuitively clear. In most cases, we use an FDR of 10%, which is a good compromise between sensitivity and specificity. We deviate from this towards a higher FDR of 20% when we want to detect broad trends but not analyze specific results in detail, and towards a lower FDR of 5% at steps which provided the genes which were critical to the downstream analyses. For further discussion of statistical cut-off choices, see notably [67].

### Aging datasets microarray analysis

For microarray datasets (both aging and caloric restriction experiments) from skeletal muscle of *M*. *musculus* and whole-body of *D*. *melanogaster*, raw Affymetrix .CEL files were downloaded from the GEO database and preprocessed using RMA normalization algorithm [68] (S1 Table). In case of multiple probes mapping to the genes on the array, the average of the probes was taken in further analysis. The annotation was used from Ensembl release 91. In order to identify the features that exhibit the most variation in the dataset, principal component analysis (PCA) was performed on the expression matrices to detect outlier samples, gender and other batches.

### Aging datasets RNA-seq analysis

For RNA-seq datasets from two model organisms, *M*. *musculus* and *C*. *elegans*, the .sra files were downloaded from the SRA database [63]. Both datasets were sequenced on Illumina HiSeq 2000 with read length 50nt. The reads were mapped to species-specific reference genomes (*M*. *musculus*: GRCm38.p5, *C*. *elegans*: WBCel235) using kallisto v0.43.1 (for index building: kallisto index–i genome.idx genome.cdna.all.fa (k-mer = 31, default option); for mapping: kallisto quant -i genome.idx–o output.file–single–l 200 –s 20 single.end.fastq.file) [69]. Both *M*. *musculus* and *C*. *elegans* had single-end RNA-seq libraries in the experiments (S1 Table). The transcript abundances were summarized at the gene-level [70]. For both species, we used GTF gene annotation files that were downloaded from Ensembl ftp site (release 91) [71]. The transcript abundances were summarized at the gene-level to lengthscaledTPMs using tximport v1.6.0 [70] and used as an input to *limma voom*. The gene-level read counts were further analyzed in R v3.4.3. The read counts were normalized by total number of all mappable reads (library size) for each gene. The *limma voom* results in a matrix of normalized gene expression values on log2 scale. The counts and normalized log2 *limma voom* expression values were used as a raw input for all the analysis. Outlier samples were checked by principal component analysis. For each species, genes that showed expression below 1 count per million (cpm < 1) in the group of replicates were excluded from downstream analysis.

### Identification of age-related differentially expressed genes

To be able to obtain differentially expressed genes from different experiments that were normalized, we had to account for the possible batches present. Since we are not aware of all the batches in the studies, we used Surrogate Variable Analysis (SVA) to correct for batches [72] in microarray data analysis. The SVA method borrows the information across gene expression levels to estimate the large-scale effects of all factors absent from the model directly from the data. After species-specific expression matrices were corrected, they served as input into linear model analysis implemented in *limma* (Affymetrix) or *limma voom* (RNA-seq) [73], for finding age-related differentially expressed genes between two extreme aging groups, young and old. Briefly, *limma* uses moderate t-statistics that includes moderated standard errors across genes, therefore effectively borrowing strength from other genes to obtain the inference about each gene. The statistical significance of putatively age-dependent genes was determined with a false discovery rate (FDR) of 10%.

### Caloric restriction datasets microarray analysis

The GEO database was used to download caloric restriction datasets (S1 Table). Only muscle tissue was available in *H*. *sapiens*, therefore we selected correspondingly muscle tissue in mouse, but whole body in fly and worm. The datasets were normalized using RMA normalization algorithm [68] (S1 Table). In case of multiple probes mapping to the genes on the array, the average of the probes was taken in further analysis. The annotation was used from Ensembl release 91. To call differentially expressed genes, we used *limma* between caloric restriction and control samples. The statistical significance of putatively age-dependent genes was determined with a false discovery rate (FDR) of 5%.

### Age group alignments between species

For deriving one-to-one orthologs, human genes were mapped to the homologs in the respective species using biomaRt v2.34.2. After detection of significant age-associated differentially expressed genes, we overlapped one-to-one orthologous genes between the species in order to observe the consistency of age groups between species. We took the *limma voom* corrected expression matrix for GTEx V6p and the expression matrices of model organisms, and selected only genes that were differentially expressed with an FDR of 5%. We then accounted for the laboratory batch effect by applying Combat on expression matrices [74].

### Gene-level analysis

To examine the relationship between aging in human and model organisms on single-gene level, we mapped one-to-one orthologous genes from human to model organisms and between the organisms downloaded from Ensembl [71]. We calculated Spearman correlations between sets of matched differentially expressed orthologous genes, between log2 fold-changes (Supplementary S2 Fig). No cutoff for fold change was used.

### Constructing homologous quadruplets and enrichment analysis

We downloaded hierarchical orthologous groups (HOGs, in further text referring to orthologous groups (OG)) across four species from the OMA (orthologous matrix analysis) database [75] at the Bilatera level (*Amphimedon queenslandica* (*Cnidaria*) was used as a metazoan outgroup), which resulted in 3232 orthologous groups. Briefly, hierarchical orthologous groups are gene families that contain orthologs (genes related by speciation) and in-paralogs (genes related by duplication) at the taxonomic level which orthologous groups were defined. The sizes of orthologous groups in this study range from 4 to 246 genes. We filtered age-related genes per orthologous group per species in order to obtain representative species-specific genes per group. The genes within orthologous group were selected according to the *P* values from differentially expression analysis [76]. We applied Bonferroni correction on each orthologous group to the differential expression *P* values in order to correct for the size of the orthologous group. We then combined the corrected differential gene expression *P* values across species using Fisher’s combined probability test generating a new *P* value from χ^2^ distribution with 2k degrees of freedom (Formula 2).

$$-2\sum_{i=1}^{k}\ln\left(P_{i}\right)∼\chi_{2k}^{2},$$ [2]

where *P_i_* is species-specific gene *P* value from differential expression analysis within a OG.

We adjusted combined Fisher *P* values for multiple testing, and filtered orthologous groups with FDR of 10% for further analysis. This resulted in 2010 and 2075 common OGs for skeletal muscle and hippocampus, respectively. In caloric restriction experiments, we detected 1962 common OGs.

We performed general GO enrichment analysis using Fisher’s test (topGO R package) on significant orthologous group genes and based on human gene set annotation to find functional enrichment of OGs in GO ‘biological process’ terms. To summarize the significantly enriched top 100 GO terms into main ones, we used the Wang GO semantic similarity method [77] that takes into account the hierarchy of gene ontology, and performed hierarchical clustering (11 clusters for skeletal muscle and 13 clusters for hippocampus, 10 clusters for caloric restriction) on the semantic matrix for both aging and caloric restriction experiments (S5 Table). The clusters were then named according to the common term of the cluster. We associated proteostasis-linked processes to GO terms associated with ‘translation’, ‘protein folding’, ‘proteasome assembly’, ‘macroautophagy’, ‘proteasome complex’, ‘endoplasmic reticulum’, ‘lysosome’ and others.

To perform the randomizations, we selected random genes from the differential expression matrices with the same number as the number of orthologous groups selected for skeletal muscle and hippocampus. The p-values associated with the random genes per species were then combined with the Fisher’s combined test. The GO enrichment analysis was performed as for the observed data with focus on the ‘biological process’ and based on the human annotation. The procedure was repeated 100 times (S6 Fig).

### Prioritization of OG gene pairs in multi-species co-expression network

We aimed to detect gene sets that are perturbed in aging in different species. We selected the genes from previously formed significant age-related OGs per species and constructed the species-specific co-expression networks by calculating Pearson correlation coefficient between age-related OGs genes. In the resulting species-specific co-expression network, nodes represent genes and edges connect genes that are above a set significant threshold from Pearson correlation calculation (*P* value < 0.05). Only positively correlated genes were taken into account, while the negatively correlated genes and genes correlating under the threshold were set to zero. Negatively correlated genes might be interesting to detect complex regulatory patterns, but are beyond the scope of this study. The cross-species network was obtained as follows **[78]**. Each co-expression link was assigned a rank within the species according to the Pearson correlation value. We then divided the species-specific ranks by the total number of OGs per tissue to normalize the ranks across the species (Formula 3, example for human, but same for other species).

$r_{n}=\frac{r_{cxh}}{N_{eog}}$ [3], where *r_n_* is normalized gene pair rank, *r_cxh_* is the rank of co-expression link in human and *N_eog_* is the number of common evolutionary orthologous groups selected for tissue.

The final gene-pair list was then obtained by integrating human, mouse, fly and worm ranked lists using robust aggregation, originally made for comparing two lists [79]. Briefly, using beta probability distribution on order statistics, we asked how probable is the co-expression link by taking into account the ranks of all four species. This method assigns a *P* value to each co-expression link in an aggregated list, indicating how much better it is ranked compared to the null model (random ordering). This yielded networks with 2887 and 3353 significant gene-pairs (edges) (*P* value < 0.001) for skeletal muscle and hippocampus, respectively.

To confirm that the integrated age-related multi-species networks are significant, we selected randomly collected genes from each species. The numbers of selected genes was the same as in the OGs. We then formed the quadruplets and performed the same integration analysis as before. We repeated the procedure 100 times, and obtained 100 randomly integrated multi-species networks (S7 Fig). In both cases, random and original analysis, the annotation was based on human.

### Clustering the integrated cross-species network

In order to identify aging-associated functional modules, we created networks containing 1142 nodes (2887 edges) in skeletal muscle and 1098 nodes (3353 edges) in hippocampus, from our prioritized gene pair list based on orthology and all edges between them. The negative logarithm (base 10) of *P* values from aggregated list was assigned as edge weights in both integrated networks. We decomposed the skeletal muscle and hippocampus integrated networks into components and the further analysis was restricted to analysis of a giant component. The giant component contained 1050 genes (nodes) in skeletal muscle and 1067 genes (nodes) in hippocampus. As before, we used human annotation. The modules within the cross-species networks of each tissue were obtained by using a multilevel community algorithm that takes into account edge weights [80] from igraph [81]. Briefly, the multilevel algorithm [82] takes into account each node as its own and assigns it to the community with which it achieves the highest contribution to modularity. To obtain Fig 4, we summarized groups of module nodes to single meta-nodes according to their multilevel-algorithm calculated module membership, and showed the inter-modular connectivity using a circular layout. We selected the modules with size greater than 10, which returned 12 modules per tissue-specific cross-species network. We checked the functional enrichment of genes within selected modules in every network using Gene Ontology through topGO R package (See Fig 4).

Moreover, we downloaded the pre-calculated file of gene-level summary statistics from 37 GWASs from the Pascal method [35]. We manually selected 22 out of 37 GWAS studies [83] (S9 Table) that are associated with metabolic, neurological, or age-related diseases. To perform enrichment of the module genes within GWAS age-related diseases categories, we selected top-ranking genes (GWAS gene score < 0.1) within each disease and formed the categories for enrichment. We ran enrichment analysis on final network modules to find disease-related modules (adjusted p-value < 0.2). The human genome was used as a background gene set.

Finally, we used Kleinberg’s hub centrality score to determine the hub genes within interested modules and observed the hub-gene neighborhood. The final genes were then selected to show their *P* value association within GWAS studies (Fig 5C, S9 Table).

### LXS and BXD mouse data

Male and female mice from those strains were fed with normal *ad libitum* diet, and median and maximum lifespan were calculated to represent longevity across strains. Microarray data as well as lifespan data were downloaded from GeneNetwork.org. Microarray data from prefrontal cortex of LXS mice was generated by Dr. Michael Miles using animals with the average age of 72 days (GN Accession: GN130). Microarray data from hippocampus of LXS mice was generated by Dr. Robert Williams using animals with the average age of 73 days (GN Accession: GN219). Microarray data from spleen of BXD mice was generated by Dr. Robert W. Williams using animals with the average age of 78 days (GN Accession: GN283). Microarray data from hippocampus of BXD mice was generated by Dr. Gerd Kempermann and Dr. Robert W. Williams using animals with the average age of 70 days (GN Accession: GN110). For enrichment analysis, genes were ranked based on their Pearson correlation coefficients with the lifespan data of the BXD strains, and Gene Set Enrichment Analysis (GSEA) was performed to find the enriched gene sets correlated with the lifespan [84].

## Supporting information

S1 Text R code for generating the figures and supplemental figures (differential expression). (PDF)

S1 Fig Detailed overview on the statistical integrative cross-species approach. A. Process-level integration. The integration is done based on the selection of the gene set families conserved across 4 species; minimum p-values from age-related differential expression analysis are used to define "age-related" genes. The p-values are combined using Fisher’s combined test. B. Network-level integration. The obtained age-related conserved genes were used for the integration of gene co-expression networks across species based on n-order statistics.
(PNG)

S2 Fig Clustering of the age-related samples between human (20-30y; 61-70y) and other species. The selected top differentially expressed genes that are orthologous between each species show alignments between the young and old samples.
(PNG)

S3 Fig The scatterplots of pairwise species comparison from single-gene level analysis. (A) Human-Mouse, (B) Human-Fly, (C) Human-Worm. No cut-off was applied. There is a weak correlation between the 1–1 orthologous genes between human and other species. This indicates that the gene-level changes in aging are species-specific.
(PNG)

S4 Fig GSEA per single-species. The panels (A-F) show the enrichments in GO BP categories (FDR < 0.20) in normal aging per species. The GSEA plots show strong enrichment in tissue-specific processes that are perturbed during aging process.
(PNG)

S5 Fig The scatterplots of p-values from single-species differential expression analysis, against the p-values integrated using Fisher’s combined test. The Fisher’s method gives more conservative than classical (per species), meaning that some genes found differentially expressed in species when combined are more significant.
(PNG)

S6 Fig Randomization on the processes-level. (PNG)

S7 Fig Proteostasis-linked processes enriched in hippocampus and caloric restriction experiments in human. The log2 GO enrichment scores are shown for both ‘biological process’ and ‘cellular component’ categories that are related to proteostasis processes.
(PNG)

S8 Fig Volcano plots of conserved gene expression in human hippocampus. Gene expression changes of the conserved genes from orthogroups enriched in main parts of proteostasis network. The genes are annotated to human genome and significance of the genes are shown on the volcano plots from human GTEx normal aging differential expression analysis of (hippocampus). The signal of loss of proteostasis in hippocampus is not that strong as in skeletal muscle (Fig 3).
(PNG)

S9 Fig The number of connections and number of modules from random networks results (100 permutations) in both skeletal muscle and hippocampus data. The conserved aging co-expression networks show low number of connections than when the integration is performed on the random genes.
(PNG)

S10 Fig **Additional interesting modules (skeletal muscle (A) on oxidation-reduction process**; hippocampus (B) on translational initiation) associated with proteostasis-linked processes and age-related GWAS. Their hub genes and genes part of the proteasome complex are shown in Fig 5C.
(PNG)

S1 Table Expression datasets used in aging and caloric restriction analysis. This table contains 2 sheets, corresponding to aging and dietary restriction experiments.
(XLSX)

S2 Table Differential expression statistics in skeletal muscle (human, mouse), hippocampus (human, mouse), whole body (fly, worm) for age-related experiments and skeletal muscle (human, mouse) and whole body (fly, worm) for dietary restriction. This table contains 6 sheets, each sheet corresponds for tissue and species. In each sheet, rows correspond to genes with no cutoffs applied. The columns provide differential expression statistics for all the samples (GTEx) and two-group comparisons (model organisms).
(XLSX)

S3 Table Overlap between the 1-to-1 conserved age-related orthologs between human and model organisms. (XLSX)

S4 Table List of orthologous genes from integrative analysis. This table contains 3 sheets, corresponding to muscle, hippocampus and dietary restriction experiments that were integrated based on orthologous groups. The columns represent name of orthogroups, combined p-values across species from Fisher’s combined probability test, original p-values from differential expression analysis per species and annotations of genes. The rows contain genes that are representative per orthologous group for each species.
(XLSX)

S5 Table Summarized clusters based on GO semantic similarity method. This table contains 3 sheets, corresponding to muscle, hippocampus and dietary restriction GO analysis. The file shows the GO enrichments and categorization to higher (more general) GO terms.
(XLSX)

S6 Table Proteostasis-linked processes enriched in 2 tissues and dietary restriction experiments. This table contains 3 sheets, corresponding to muscle, hippocampus and dietary restriction GO analysis for proteostasis-linked processes.
(XLSX)

S7 Table Significant conserved genes from human GTEx in proteostasis quality network for skeletal muscle and hippocampus. This table contains 6 sheets for each part of the protein quality network (macroautophagy, translation and proteasome complex) per tissue.
(XLSX)

S8 Table Summary of the statistics from network analysis. This table contains 5 sheets of the information about the sizes of the all modules and GO and GWAS enrichments in each tissue for proteostasis-linked modules.
(XLSX)

S9 Table Summary of mapping the GWAS traits for selected modules. This table contains the gene-level p-values from the PASCAL tool for the heatmap of Fig 5C for selected 22 GWAS age-related studies.
(XLSX)

## References

[1] Jones OR, Scheuerlein A, Salguero-Gómez R, Camarda CG, Schaible R, Casper BB, et al Diversity of ageing across the tree of life. Nature. 2014;505: 169–173. 10.1038/nature12789 24317695 PMC4157354

[2] Rappuoli R, Mandl CW, Black S, De Gregorio E. Vaccines for the twenty-first century society. Nat Rev Immunol. Nature Publishing Group; 2011;11: nri3085 10.1038/nri3085 22051890 PMC7098427

[3] Greene VW. Personal hygiene and life expectancy improvements since 1850: Historic and epidemiologic associations. Am J Infect Control. Mosby; 2001;29: 203–206. 10.1067/mic.2001.115686 11486254

[4] Mattison JA, Colman RJ, Beasley TM, Allison DB, Kemnitz JW, Roth GS, et al Caloric restriction improves health and survival of rhesus monkeys. Nat Commun. Nature Publishing Group; 2017;8: 14063 10.1038/ncomms14063 28094793 PMC5247583

[5] Bass TM, Grandison RC, Wong R, Martinez P, Partridge L, Piper MDW. Optimization of Dietary Restriction Protocols in Drosophila. J Gerontol Ser A. 2007;62: 1071–1081. 10.1093/gerona/62.10.1071 17921418 PMC4335187

[6] Lee GD, Wilson MA, Zhu M, Wolkow CA, De Cabo R, Ingram DK, et al Dietary deprivation extends lifespan in Caenorhabditis elegans. Aging Cell. 2006;5: 515–524. 10.1111/j.1474-9726.2006.00241.x 17096674 PMC2546582

[7] Selman C, Hempenstall S. Evidence of a metabolic memory to early-life dietary restriction in male C57BL/6 mice. Longev Heal. 2012;1: 2 10.1186/2046-2395-1-2 24764508 PMC3886256

[8] Plank M, Wuttke D, van Dam S, Clarke SA, de Magalhaes JP. A meta-analysis of caloric restriction gene expression profiles to infer common signatures and regulatory mechanisms. Mol Biosyst. 2012;8: 1339–1349. 10.1039/c2mb05255e 22327899

[9] López-Otín C, Blasco MA, Partridge L, Serrano M, Kroemer G. The hallmarks of aging. Cell. 2013;153 10.1016/j.cell.2013.05.039 23746838 PMC3836174

[10] Smith ED, Tsuchiya M, Fox LA, Dang N, Hu D, Kerr EO, et al Quantitative evidence for conserved longevity pathways between divergent eukaryotic species. Genome Res. 2008;18: 564–570. 10.1101/gr.074724.107 18340043 PMC2279244

[11] Fushan AA, Turanov AA, Lee S-G, Kim EB, Lobanov A V, Yim SH, et al Gene expression defines natural changes in mammalian lifespan. Aging Cell. 2015;14: 352–65. 10.1111/acel.12283 25677554 PMC4406664

[12] Zahn JM, Sonu R, Vogel H, Crane E, Mazan-Mamczarz K, Rabkin R, et al Transcriptional Profiling of Aging in Human Muscle Reveals a Common Aging Signature. PLoS Genet. McGraw-Hill; 2006;2: e115 10.1371/journal.pgen.0020115 16789832 PMC1513263

[13] Rogina B. For the special issue: Aging studies in Drosophila melanogaster. Exp Gerontol. 2011;46: 317–319. 10.1016/j.exger.2010.09.001 20828603 PMC3008287

[14] Morrow G, Tanguay RM. Mitochondria and ageing in Drosophila. Biotechnol J. 2008;3: 728–739. 10.1002/biot.200800015 18446867

[15] Wilkinson DS, Taylor RC, Dillin A. Chapter 12—Analysis of Aging in Caenorhabditis elegans. In: Rothman JH, Singson A, editors. Methods in Cell Biology. Academic Press; 2012 pp. 353–381. 10.1016/B978-0-12-394620-1.00012-6 22226530

[16] Lezzerini M, Smith RL, Budovskaya Y. Developmental drift as a mechanism for aging: lessons from nematodes. Biogerontology. 2013;14: 693–701. 10.1007/s10522-013-9462-3 24122213

[17] Sapieha P, Mallette FA. Cellular Senescence in Postmitotic Cells: Beyond Growth Arrest. Trends Cell Biol. 2018;28: 595–607. 10.1016/j.tcb.2018.03.003 29704982

[18] Jarman SN, Polanowski AM, Faux CE, Robbins J, Paoli‐Iseppi RD, Bravington M, et al Molecular biomarkers for chronological age in animal ecology. Mol Ecol. 2015;24: 4826–4847. 10.1111/mec.13357 26308242

[19] Fontana L, Partridge L, Longo VD. Extending Healthy Life Span—From Yeast to Humans. Science. 2010;328: 321–326. 10.1126/science.1172539 20395504 PMC3607354

[20] Hasin Y, Seldin M, Lusis A. Multi-omics approaches to disease. Genome Biol. 2017;18: 83 10.1186/s13059-017-1215-1 28476144 PMC5418815

[21] Baumgart M, Priebe S, Groth M, Hartmann N, Menzel U, Pandolfini L, et al Longitudinal RNA-Seq Analysis of Vertebrate Aging Identifies Mitochondrial Complex I as a Small-Molecule-Sensitive Modifier of Lifespan. Cell Syst. Elsevier; 2016;2: 122–132. 10.1016/j.cels.2016.01.014 27135165

[22] van Dam S, Võsa U, van der Graaf A, Franke L, de Magalhães JP. Gene co-expression analysis for functional classification and gene–disease predictions. Brief Bioinform. 2017; bbw139 10.1093/bib/bbw139 28077403 PMC6054162

[23] Xue H, Xian B, Dong D, Xia K, Zhu S, Zhang Z, et al A modular network model of aging. Mol Syst Biol. 2007;3: 147 10.1038/msb4100189 18059442 PMC2174624

[24] Mele M, Ferreira PG, Reverter F, DeLuca DS, Monlong J, Sammeth M, et al The human transcriptome across tissues and individuals. Science. 2015;348: 660–665. 10.1126/science.aaa0355 25954002 PMC4547472

[25] Lonsdale J, Thomas J, Salvatore M, Phillips R, Lo E, Shad S, et al The Genotype-Tissue Expression (GTEx) project. Nat Genet. 2013;45: 580–585. 10.1038/ng.2653 23715323 PMC4010069

[26] Marzetti E, Leeuwenburgh C. Skeletal muscle apoptosis, sarcopenia and frailty at old age. Exp Gerontol. 2006;41: 1234–1238. 10.1016/j.exger.2006.08.011 17052879

[27] Driscoll I, Hamilton DA, Petropoulos H, Yeo RA, Brooks WM, Baumgartner RN, et al The Aging Hippocampus: Cognitive, Biochemical and Structural Findings. Cereb Cortex. Oxford University Press; 2003;13: 1344–1351. 10.1093/cercor/bhg081 14615299

[28] de Magalhães JP, Curado J, Church GM. Meta-analysis of age-related gene expression profiles identifies common signatures of aging. Bioinforma Oxf Engl. 2009;25: 875–81. 10.1093/bioinformatics/btp073 19189975 PMC2732303

[29] Yang J, Huang T, Petralia F, Long Q, Zhang B, Argmann C, et al Synchronized age-related gene expression changes across multiple tissues in human and the link to complex diseases. Sci Rep. 2015;5: 15145 10.1038/srep15145 26477495 PMC4609956

[30] Flurkey K, M. Currer J, Harrison DE. Chapter 20 –Mouse Models in Aging Research. The Mouse in Biomedical Research. 2007 pp. 637–672. 10.1016/B978-012369454-6/50074-1

[31] Fushan AA, Turanov AA, Lee SG, Kim EB, Lobanov A V., Yim SH, et al Gene expression defines natural changes in mammalian lifespan. Aging Cell. 2015;14: 352–365. 10.1111/acel.12283 25677554 PMC4406664

[32] Gene Ontology Consortium T, Ashburner M, Ball CA, Blake JA, Botstein D, Butler H, et al Gene Ontology: tool for the unification of biology. Nat Genet. 2000;25: 25–29. 10.1038/75556 10802651 PMC3037419

[33] The Gene Ontology Consortium. Expansion of the Gene Ontology knowledgebase and resources. Nucleic Acids Res. 2017;45: D331–D338. 10.1093/nar/gkw1108 27899567 PMC5210579

[34] Lanna A, Gomes DCO, Muller-Durovic B, McDonnell T, Escors D, Gilroy DW, et al A sestrin-dependent Erk–Jnk–p38 MAPK activation complex inhibits immunity during aging. Nat Immunol. Nature Research; 2017;18: 354–363. 10.1038/ni.3665 28114291 PMC5321575

[35] Lamparter D, Marbach D, Rueedi R, Kutalik Z, Bergmann S, Kutalik Z. Fast and Rigorous Computation of Gene and Pathway Scores from SNP-Based Summary Statistics. Listgarten J, editor. PLOS Comput Biol. Public Library of Science; 2016;12: e1004714 10.1371/journal.pcbi.1004714 26808494 PMC4726509

[36] Andreux PA, Wiliams EG, Koutnikova H, Houtkooper RH, Champy M-F, Hugues H, et al Systems Genetics of Metabolism: The Use of the BXD Murine Reference Panel for Multiscalar Integration of Traits. Cell. Cell Press; 2012;150: 1287–1299. 10.1016/j.cell.2012.08.012 22939713 PMC3604687

[37] Li H, Wang X, Rukina D, Huang Q, Lin T, Sorrentino V, et al An Integrated Systems Genetics and Omics Toolkit to Probe Gene Function. Cell Syst. 2018;6: 90–102.e4. 10.1016/j.cels.2017.10.016 29199021

[38] Liao CY, Rikke BA, Johnson TE, Diaz V, Nelson JF. Genetic variation in the murine lifespan response to dietary restriction: From life extension to life shortening. Aging Cell. 2010;9: 92–95. 10.1111/j.1474-9726.2009.00533.x 19878144 PMC3476836

[39] Gems D, Partridge L. Genetics of Longevity in Model Organisms: Debates and Paradigm Shifts. Annu Rev Physiol. 2013;75: 621–44. 10.1146/annurev-physiol-030212-183712 23190075

[40] Davis EJ, Lobach I, Dubal DB. Female XX sex chromosomes increase survival and extend lifespan in aging mice. Aging Cell. 0: e12871 10.1111/acel.12871 30560587 PMC6351820

[41] Austad SN, Fischer KE. Sex Differences in Lifespan. Cell Metab. 2016;23: 1022–1033. 10.1016/j.cmet.2016.05.019 27304504 PMC4932837

[42] Akker EB, Passtoors WM, Jansen R, Zwet EW, Goeman JJ, Hulsman M, et al Meta‐analysis on blood transcriptomic studies identifies consistently coexpressed protein–protein interaction modules as robust markers of human aging. Aging Cell. 2014;13: 216–225. 10.1111/acel.12160 24119000 PMC4331790

[43] Budovsky A, Abramovich A, Cohen R, Chalifa-Caspi V, Fraifeld V. Longevity network: Construction and implications. 2006; 10.1016/j.mad.2006.11.018 17116322

[44] Southworth LK, Owen AB, Kim SK. Aging mice show a decreasing correlation of gene expression within genetic modules. PLoS Genet. 2009;5 10.1371/journal.pgen.1000776 20019809 PMC2788246

[45] Fernandes M, Wan C, Tacutu R, Barardo D, Rajput A, Wang J, et al Systematic analysis of the gerontome reveals links between aging and age-related diseases. Hum Mol Genet. Oxford University Press; 2016;25: ddw307 10.1093/hmg/ddw307 28175300 PMC5418736

[46] Mcdaid AF, Joshi PK, Porcu E, Komljenovic A, Li H, Sorrentino V, et al ARTICLE Bayesian association scan reveals loci associated with human lifespan and linked biomarkers. Nat Commun. 2017;8 10.1038/ncomms15842 28748955 PMC5537485

[47] Finkel T. The metabolic regulation of aging. Nat Med. 2015;21: 1416–1423. 10.1038/nm.3998 26646498

[48] Roy AK, Oh T, Rivera O, Mubiru J, Song CS, Chatterjee B. Impacts of transcriptional regulation on aging and senescence. Ageing Res Rev. 2002;1: 367–80. 12067592 10.1016/s1568-1637(02)00006-5

[49] Steffen KK, Dillin A. A Ribosomal Perspective on Proteostasis and Aging. 2016; 10.1016/j.cmet.2016.05.013 27304502

[50] Labbadia J, Morimoto RI. The Biology of Proteostasis in Aging and Disease. Annu Rev Biochem. Annual Reviews; 2015;84: 435–464. 10.1146/annurev-biochem-060614-033955 25784053 PMC4539002

[51] Kaushik S, Cuervo AM. Proteostasis and aging. Nat Med. Nature Research; 2015;21: 1406–1415. 10.1038/nm.4001 26646497

[52] Rodriguez KA, Edrey YH, Osmulski P, Gaczynska M, Buffenstein R. Altered Composition of Liver Proteasome Assemblies Contributes to Enhanced Proteasome Activity in the Exceptionally Long-Lived Naked Mole-Rat. Brodsky JL, editor. PLoS ONE. 2012;7: e35890 10.1371/journal.pone.0035890 22567116 PMC3342291

[53] Pyo J-O, Yoo S-M, Ahn H-H, Nah J, Hong S-H, Kam T-I, et al Overexpression of Atg5 in mice activates autophagy and extends lifespan. Nat Commun. Nature Publishing Group; 2013;4: ncomms3300 10.1038/ncomms3300 23939249 PMC3753544

[54] Tian X, Seluanov A, Gorbunova V. Molecular Mechanisms Determining Lifespan in Short- and Long-Lived Species. Trends Endocrinol Metab TEM. Elsevier; 2017;28: 722–734. 10.1016/j.tem.2017.07.004 28888702 PMC5679293

[55] Bass TM, Grandison RC, Wong R, Martinez P, Partridge L, Piper MDW. Europe PMC Funders Group Optimization of Dietary Restriction Protocols in Drosophila. 2015;62: 1071–1081. 10.1093/gerona/62.10.1071 PMC4335187 17921418

[56] Hahn O, Grönke S, Stubbs TM, Ficz G, Hendrich O, Krueger F, et al Dietary restriction protects from age-associated DNA methylation and induces epigenetic reprogramming of lipid metabolism. Genome Biol. BioMed Central; 2017;18: 56 10.1186/s13059-017-1187-1 28351387 PMC5370449

[57] Walther DM, Kasturi P, Zheng M, Pinkert S, Vecchi G, Ciryam P, et al Erratum: Widespread Proteome Remodeling and Aggregation in Aging C. elegans (Cell (2015) 161(4) (919–932) (S0092867415003207)(10.1016/j.cell.2015.03.032). Cell. Elsevier Inc.; 2017;168: 944 10.1016/j.cell.2016.12.041 25957690 PMC4643853

[58] D ‘amico D, Sorrentino V, Auwerx J. Cytosolic Proteostasis Networks of the Mitochondrial Stress Response. 2017; 10.1016/j.tibs.2017.05.002 28579074

[59] Schmidt M, Finley D. Regulation of proteasome activity in health and disease. Biochim Biophys Acta. NIH Public Access; 2014;1843: 13–25. 10.1016/j.bbamcr.2013.08.012 23994620 PMC3858528

[60] Depuydt G, Xie F, Petyuk VA, Shanmugam N, Smolders A, Dhondt I, et al Reduced insulin/insulin-like growth factor-1 signaling and dietary restriction inhibit translation but preserve muscle mass in Caenorhabditis elegans. Mol Cell Proteomics MCP. American Society for Biochemistry and Molecular Biology; 2013;12: 3624–39. 10.1074/mcp.M113.027383 24002365 PMC3861712

[61] Chondrogianni N, Georgila K, Kourtis N, Tavernarakis N, Gonos ES. 20S proteasome activation promotes life span extension and resistance to proteotoxicity in Caenorhabditis elegans. FASEB J Off Publ Fed Am Soc Exp Biol. Federation of American Societies for Experimental Biology; 2015;29: 611–22. 10.1096/fj.14-252189 25395451 PMC4314225

[62] Barrett T, Wilhite SE, Ledoux P, Evangelista C, Kim IF, Tomashevsky M, et al NCBI GEO: Archive for functional genomics data sets—Update. Nucleic Acids Res. 2013;41: 991–995. 10.1093/nar/gks1193 23193258 PMC3531084

[63] Leinonen R, Sugawara H, Shumway M, International Nucleotide Sequence Database Collaboration. The sequence read archive. Nucleic Acids Res. Oxford University Press; 2011;39: D19–21. 10.1093/nar/gkq1019 21062823 PMC3013647

[64] Stegle O, Parts L, Piipari M, Winn J, Durbin R. Using probabilistic estimation of expression residuals (PEER) to obtain increased power and interpretability of gene expression analyses. Nat Protoc. Europe PMC Funders; 2012;7: 500–7. 10.1038/nprot.2011.457 22343431 PMC3398141

[65] Lee JJ, Wedow R, Okbay A, Kong E, Maghzian O, Zacher M, et al Gene discovery and polygenic prediction from a genome-wide association study of educational attainment in 1.1 million individuals. Nat Genet. 2018;50: 1112 10.1038/s41588-018-0147-3 30038396 PMC6393768

[66] Pers TH, Karjalainen JM, Chan Y, Westra H-J, Wood AR, Yang J, et al Biological interpretation of genome-wide association studies using predicted gene functions. Nat Commun. 2015;6: 5890 10.1038/ncomms6890 25597830 PMC4420238

[67] Amrhein V, Greenland S, McShane B. Scientists rise up against statistical significance. Nature. 2019;567: 305 10.1038/d41586-019-00857-9 30894741

[68] Irizarry RA, Bolstad BM, Collin F, Cope LM, Hobbs B, Speed TP. Summaries of Affymetrix GeneChip probe level data. Nucleic Acids Res. 2003;31: e15 10.1093/nar/gng015 12582260 PMC150247

[69] Bray NL, Pimentel H, Melsted P, Pachter L. Near-optimal probabilistic RNA-seq quantification. Nat Biotechnol. 2016;34: 525–527. 10.1038/nbt.3519 27043002

[70] Soneson C, Love MI, Robinson MD. Differential analyses for RNA-seq: transcript-level estimates improve gene-level inferences. F1000Research. 2015;4: 1521 10.12688/f1000research.7563.2 26925227 PMC4712774

[71] Aken BL, Ayling S, Barrell D, Clarke L, Curwen V, Fairley S, et al The Ensembl gene annotation system. Database. Oxford University Press; 2016;2016: baw093 10.1093/database/baw093 27337980 PMC4919035

[72] Leek JT, Storey JD. Capturing heterogeneity in gene expression studies by surrogate variable analysis. PLoS Genet. 2007;3: 1724–1735. 10.1371/journal.pgen.0030161 17907809 PMC1994707

[73] Law CW, Chen Y, Shi W, Smyth GK. voom: precision weights unlock linear model analysis tools for RNA-seq read counts. Genome Biol. 2014;15: R29 10.1186/gb-2014-15-2-r29 24485249 PMC4053721

[74] Leek JT, Johnson WE, Parker HS, Jaffe AE, Storey JD. The sva package for removing batch effects and other unwanted variation in high-throughput experiments. Bioinformatics. Oxford University Press; 2012;28: 882–883. 10.1093/bioinformatics/bts034 22257669 PMC3307112

[75] Altenhoff AM, kunca N, Glover N, Train C-M, Sueki A, Pili ota I, et al The OMA orthology database in 2015: function predictions, better plant support, synteny view and other improvements. Nucleic Acids Res. Oxford University Press; 2015;43: D240–D249. 10.1093/nar/gku1158 25399418 PMC4383958

[76] Rittschof CC, Bukhari SA, Sloofman LG, Troy JM, Caetano-Anollés D, Cash-Ahmed A, et al Neuromolecular responses to social challenge: Common mechanisms across mouse, stickleback fish, and honey bee. Proc Natl Acad Sci. 2014;111: 17929–17934. 10.1073/pnas.1420369111 25453090 PMC4273386

[77] Wang JZ, Du Z, Payattakool R, Yu PS, Chen C-F. A new method to measure the semantic similarity of GO terms. Bioinformatics. 2007;23: 1274–1281. 10.1093/bioinformatics/btm087 17344234

[78] Stuart JM, Segal E, Koller D, Kim SK. A Gene-Coexpression Network for Global Discovery of Conserved Genetic Modules. Science. 2003;302: 249–255. 10.1126/science.1087447 12934013

[79] Kolde R, Laur S, Adler P, Vilo J. Robust rank aggregation for gene list integration and meta-analysis. Bioinformatics. Oxford University Press; 2012;28: 573–580. 10.1093/bioinformatics/btr709 22247279 PMC3278763

[80] Yang Z, Algesheimer R, Tessone CJ. A Comparative Analysis of Community Detection Algorithms on Artificial Networks. Sci Rep. Nature Publishing Group; 2016;6: 30750 10.1038/srep30750 27476470 PMC4967864

[81] Csárdi G, Nepusz T. The igraph software package for complex network research. InterJournal Complex Syst. 2006; 1695.

[82] Blondel VD, Guillaume J-L, Lambiotte R, Lefebvre E. Fast unfolding of communities in large networks. J Stat Mech Theory Exp. 2008;2008: P10008 10.1088/1742-5468/2008/10/P10008

[83] Marbach D, Lamparter D, Quon G, Kellis M, Kutalik Z, Bergmann S. Tissue-specific regulatory circuits reveal variable modular perturbations across complex diseases. Nat Methods. 2016;13: 1–44. 10.1038/nmeth.3799 26950747 PMC4967716

[84] Subramanian A, Tamayo P, Mootha VK, Mukherjee S, Ebert BL, Gillette M a, et al Gene set enrichment analysis: a knowledge-based approach for interpreting genome-wide expression profiles. Proc Natl Acad Sci U S A. 2005;102: 15545–50. 10.1073/pnas.0506580102 16199517 PMC1239896

