# Supplementary figures and images for: Cross-species functional modules link proteostasis to human normal aging
Source: PLoS Comput Biol. 2019 Jul 3;15(7):e1007162. doi: 10.1371/journal.pcbi.1007162 (PMC6634426; doi:10.1371/journal.pcbi.1007162)

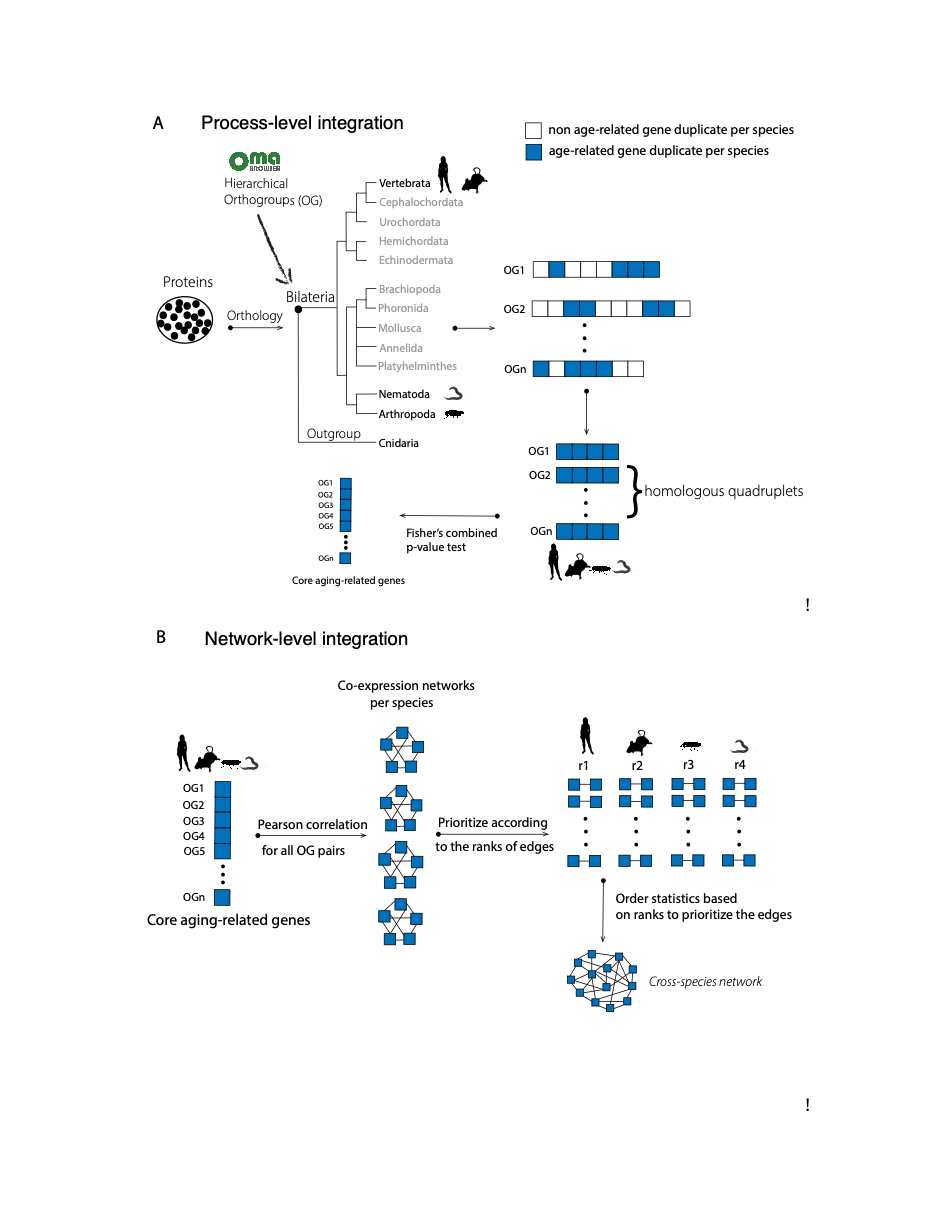

Supplement: S1 Fig — A. Process-level integration. The integration is done based on the selection of the gene set families conserved across 4 species; minimum p-values from age-related differential expression analysis are used to define "age-related" genes. The p-values are combined using Fisher’s combined test. B. Network-level integration. The obtained age-related conserved genes were used for the integration of gene co-expression networks across species based on n-order statistics. (PNG) [file pcbi.1007162.s002.png]

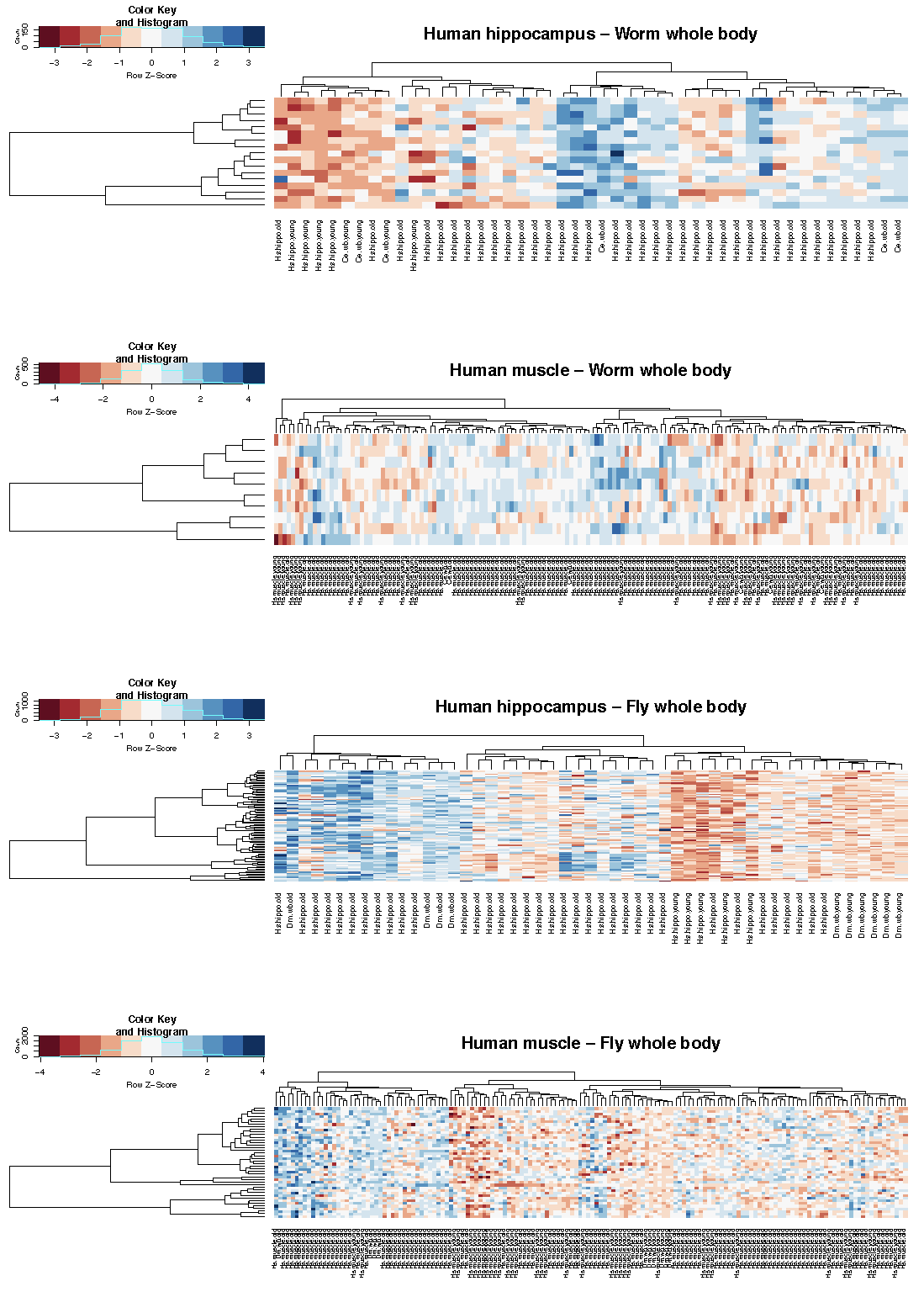

Supplement: S2 Fig — The selected top differentially expressed genes that are orthologous between each species show alignments between the young and old samples. (PNG) [file pcbi.1007162.s003.png]

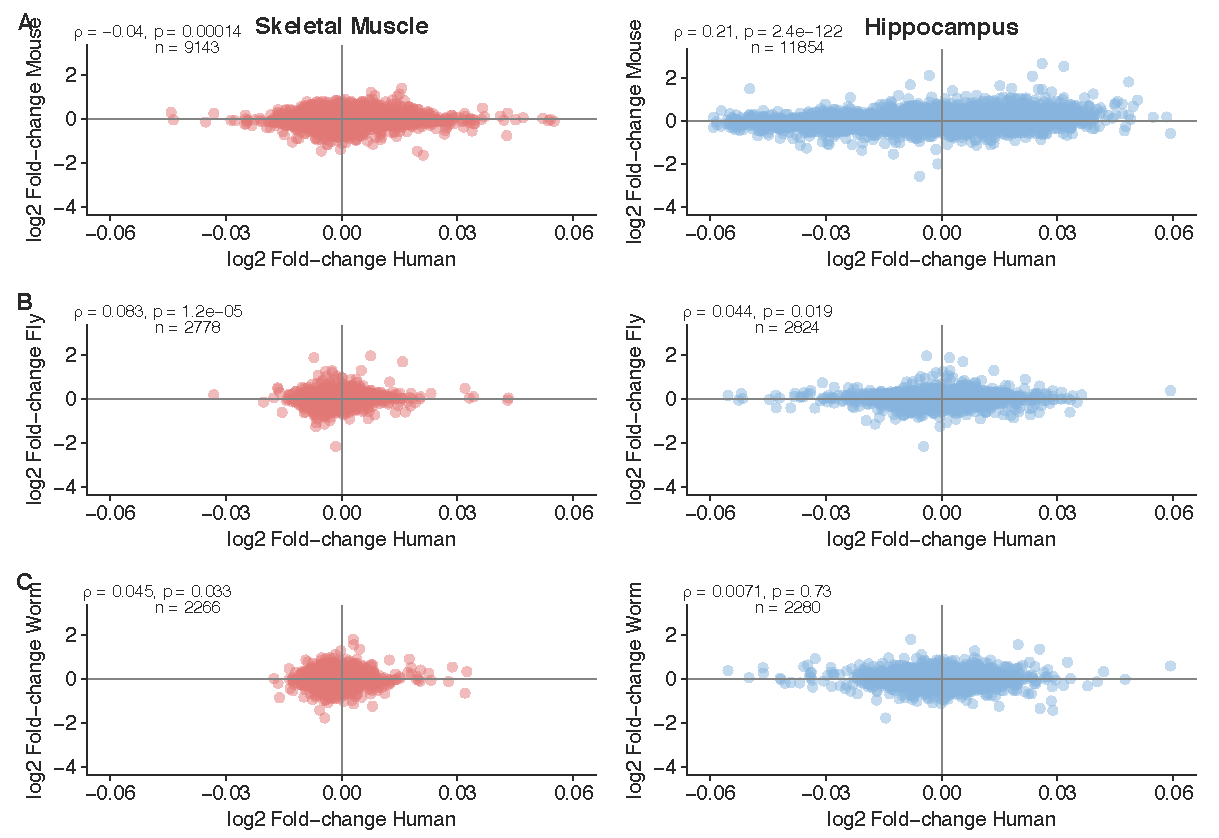

Supplement: S3 Fig — (A) Human-Mouse, (B) Human-Fly, (C) Human-Worm. No cut-off was applied. There is a weak correlation between the 1–1 orthologous genes between human and other species. This indicates that the gene-level changes in aging are species-specific. (PNG) [file pcbi.1007162.s004.png]

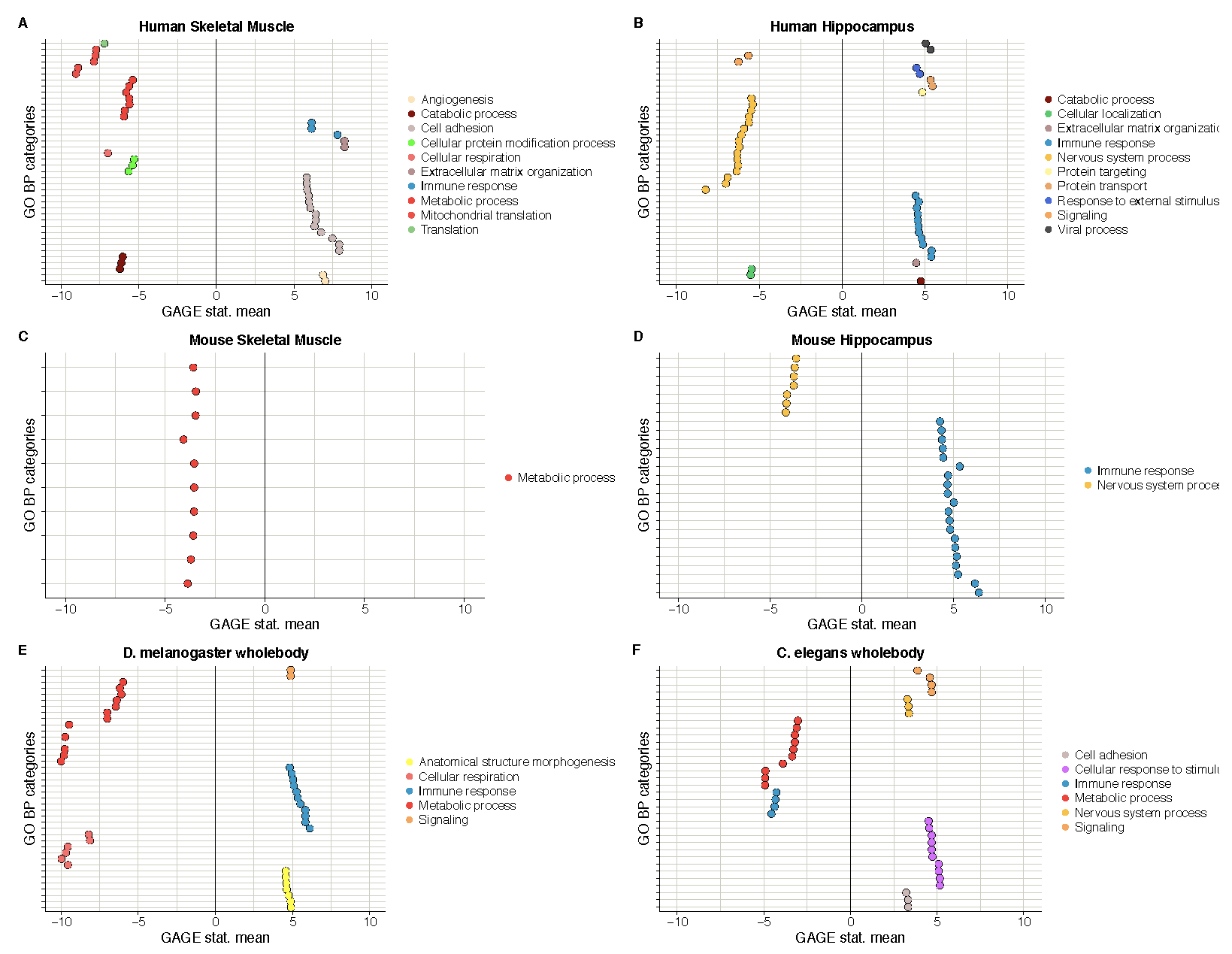

Supplement: S4 Fig — The panels (A-F) show the enrichments in GO BP categories (FDR < 0.20) in normal aging per species. The GSEA plots show strong enrichment in tissue-specific processes that are perturbed during aging process. (PNG) [file pcbi.1007162.s005.png]

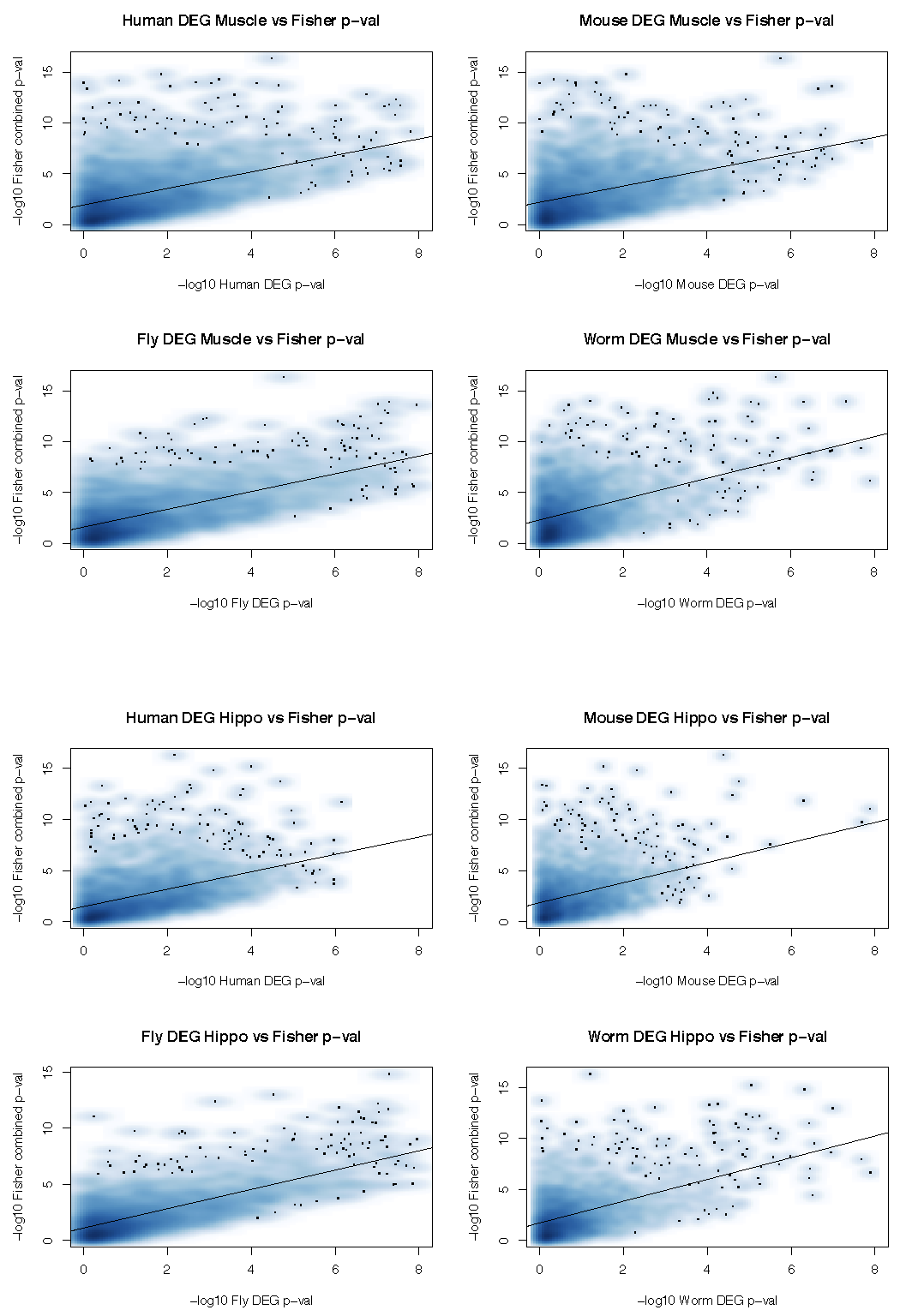

Supplement: S5 Fig — The Fisher’s method gives more conservative than classical (per species), meaning that some genes found differentially expressed in species when combined are more significant. (PNG) [file pcbi.1007162.s006.png]

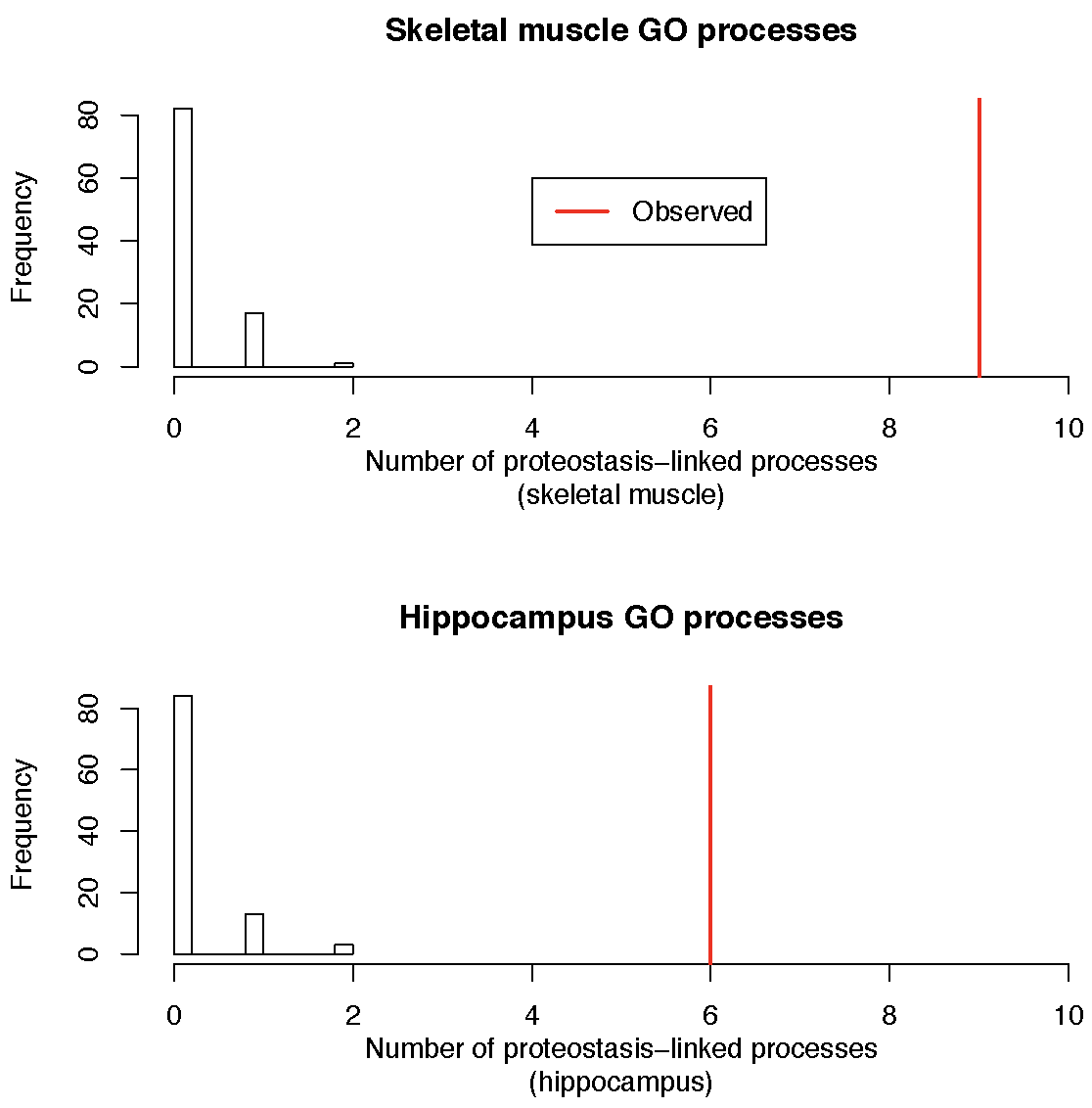

Supplement: S6 Fig — (PNG) [file pcbi.1007162.s007.png]

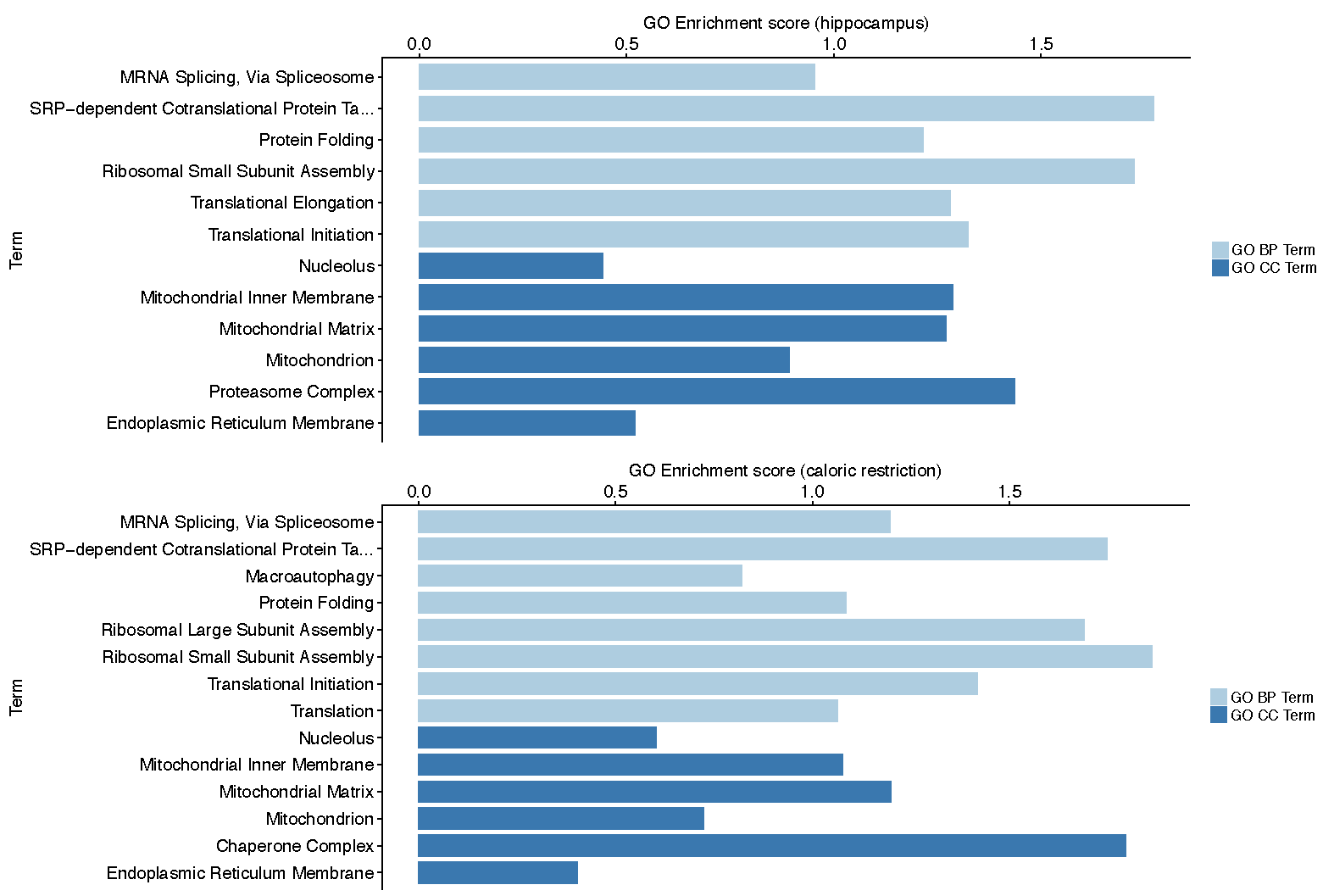

Supplement: S7 Fig — The log2 GO enrichment scores are shown for both ‘biological process’ and ‘cellular component’ categories that are related to proteostasis processes. (PNG) [file pcbi.1007162.s008.png]

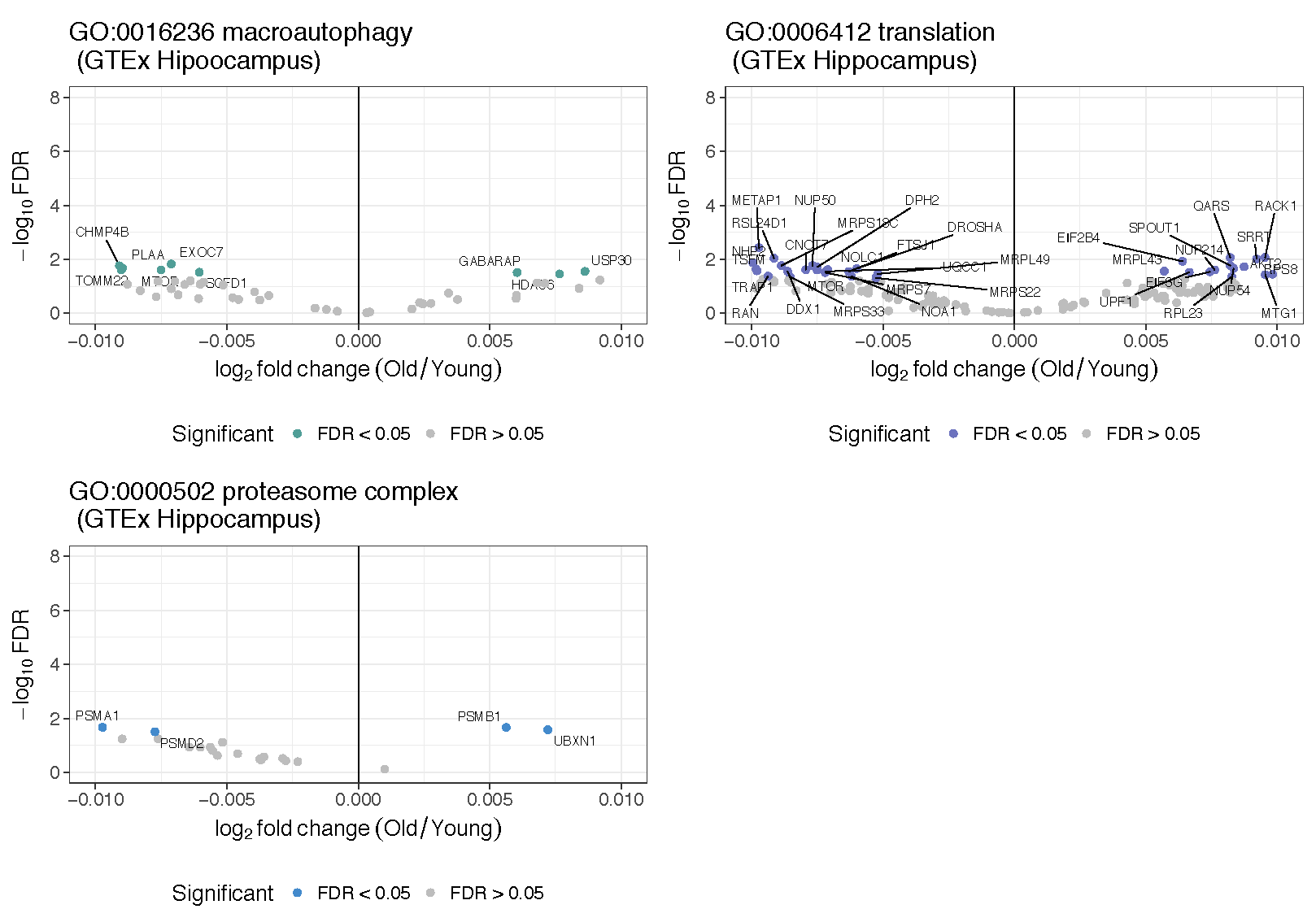

Supplement: S8 Fig — Gene expression changes of the conserved genes from orthogroups enriched in main parts of proteostasis network. The genes are annotated to human genome and significance of the genes are shown on the volcano plots from human GTEx normal aging differential expression analysis of (hippocampus). The signal of loss of proteostasis in hippocampus is not that strong as in skeletal muscle (Fig 3). (PNG) [file pcbi.1007162.s009.png]

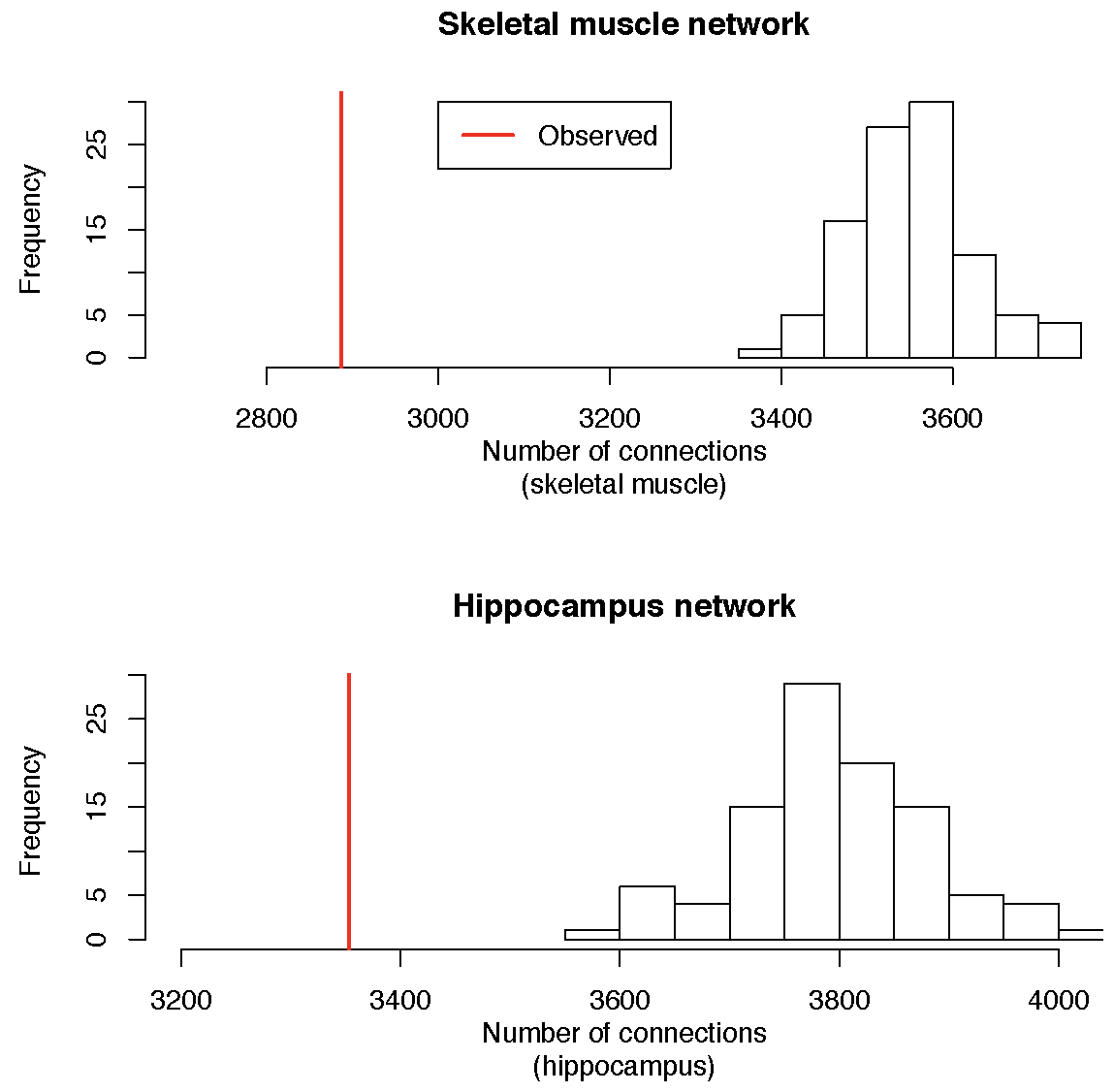

Supplement: S9 Fig — The conserved aging co-expression networks show low number of connections than when the integration is performed on the random genes. (PNG) [file pcbi.1007162.s010.png]

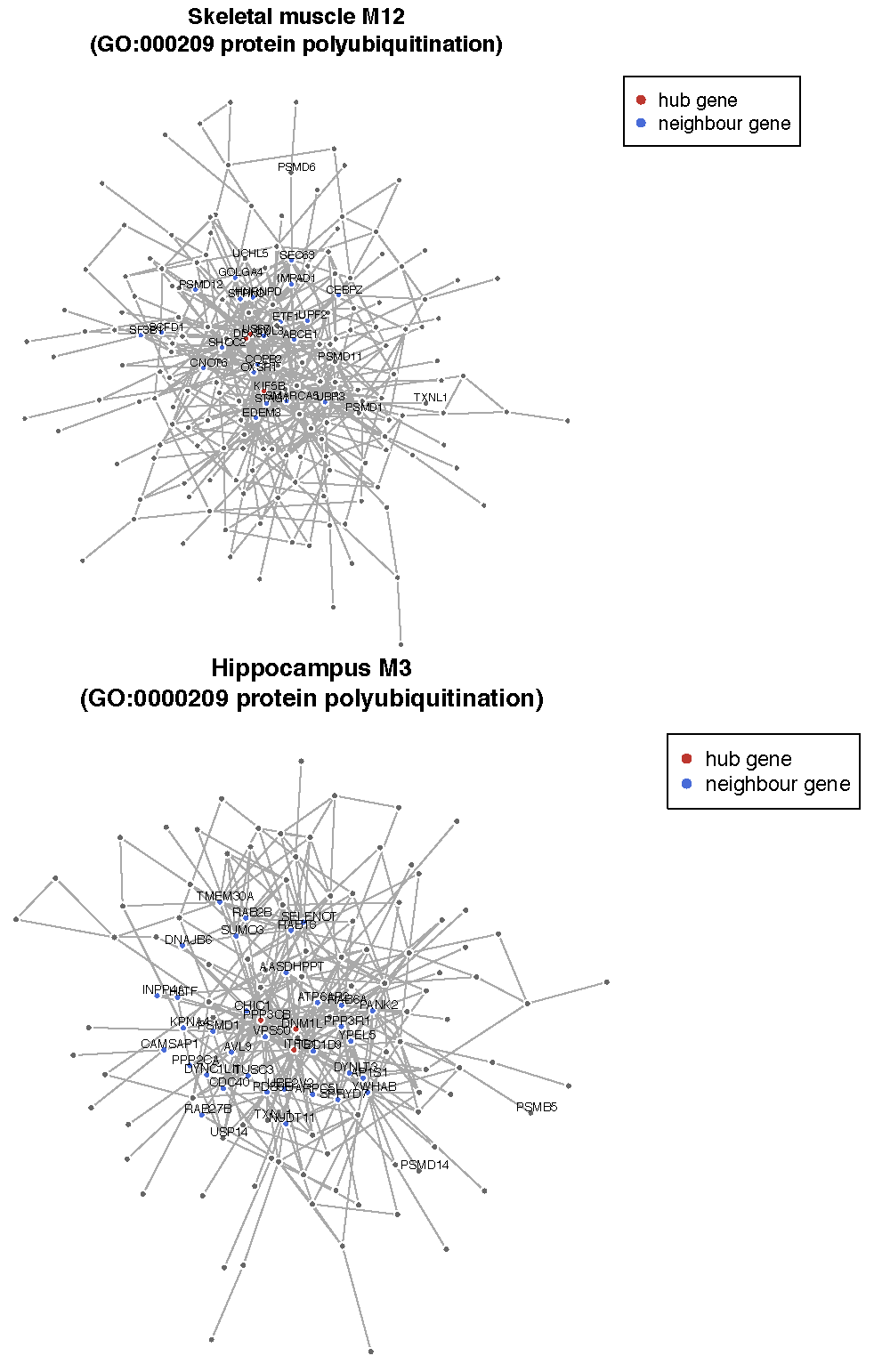

Supplement: S10 Fig — Additional interesting modules (skeletal muscle (A) on oxidation-reduction process; hippocampus (B) on translational initiation) associated with proteostasis-linked processes and age-related GWAS. Their hub genes and genes part of the proteasome complex are shown in Fig 5C. (PNG) [file pcbi.1007162.s011.png]
